# Equal long‐term care for equal needs with universal and comprehensive coverage? An assessment using Dutch administrative data

**DOI:** 10.1002/hec.3994

**Published:** 2020-01-20

**Authors:** Marianne Tenand, Pieter Bakx, Eddy van Doorslaer

**Affiliations:** ^1^ Erasmus School of Health Policy & Management (ESHPM) Erasmus University Rotterdam Rotterdam The Netherlands; ^2^ Erasmus School of Health Policy & Management (ESHPM), Erasmus School of Economics (ESE) Erasmus University Rotterdam Rotterdam The Netherlands

**Keywords:** long‐term care, equity in care use, horizontal equity, socioeconomic inequality

## Abstract

The Netherlands is one of the few countries that offer generous universal public coverage of long‐term care (LTC). Does this ensure that the Dutch elderly with similar care needs receive similar LTC, irrespective of their income? In contrast with previous studies of inequity in care use that relied on a statistically derived variable of needs, our paper exploits a readily available, administrative measure of LTC needs stemming from the eligibility assessment organized by the Dutch LTC assessment agency. Using exhaustive administrative register data on 616,934 individuals aged 60 and older eligible for public LTC, we find a substantial pro‐poor concentration of LTC use that is only partially explained by poorer individuals' greater needs. Among those eligible for institutional care, higher‐income individuals are more likely to use—less costly—home care. This pattern may be explained by differences in preferences, but also by their higher copayments for nursing homes and by greater feasibility of home‐based LTC arrangements for richer elderly. At face value, our findings suggest that the Dutch LTC insurance “overshoots” its target to ensure that LTC is accessible to poorer elderly. Yet, the implications depend on the origins of the difference and one's normative stance.

## 1 INTRODUCTION

In view of the current rate of population aging, Organisation for Economic Co‐operation and Development (OECD) countries expect the number of elderly requiring assistance in activities of daily living to rise sharply in the coming decades. As all developed countries have introduced schemes for financing the use of long‐term care (LTC hereafter) by the disabled elderly, public spending on LTC is projected to soar from 1.4% of gross domestic product (GDP) in 2014 to 4.3% by 2060 in Europe (Economic Policy Committee, 2015). On top of concerns relating to its sustainability, this increase raises another critical policy question: Does public funding ensure an equitable distribution of LTC? Although the cross‐country variation in the organization and financing of LTC systems is large (Colombo et al., 2011; Muir, 2017), empirical evidence on the ability of existing policies to ensure that all individuals have access to adequate LTC regardless of their socioeconomic resources is still scarce. Yet, such evidence is essential to inform the public debate, as many countries are currently reforming their LTC systems, either to broaden coverage or to reign in increasing spending.

The aim of our paper is twofold. We first assess whether LTC is allocated according to care needs in the Netherlands, irrespective of income—that is to say, whether LTC use is distributed according to the traditional principle of socioeconomic horizontal equity. In a second step, we discuss the normative and policy implications of our results, in terms of the ability of the Dutch public insurance to ensure equitable LTC use.

Studying inequalities in the Dutch LTC system is highly relevant, as the Netherlands stands out as a model from an international perspective. With the second highest spending in terms of GDP of all OECD countries (4.3% of GDP in 2014; Organisation for Economic Co‐operation and Development [OECD], 2017), the Dutch LTC system provides universal and comprehensive coverage and has demonstrated an ability to insure the elderly against high out‐of‐pocket payments on home care and institutional care (Bakx et al., 2015; Mot, 2010; Schut, Sorbe, & Hoj, 2013). Most importantly, ability to pay is *not* one of the criteria taken into account for eligibility for publicly subsidized LTC. 1
The criteria are listed in the decree on care‐related assessments (*Het Zorgindicatiebesluit*, 1997), Article 6. As a result, the system is often perceived as leaving little room for inequalities, as stated by Mot (2010, p. 66): “While the system in the Netherlands is not completely egalitarian, it is not too far from it.” However, empirical support for this claim is scant.

Another distinctive feature of the Netherlands is the quality of available data on LTC. We exploit exhaustive administrative registers providing information both on the eligibility decisions made by the Dutch agency in charge of assessing the needs of all individuals applying to LTC benefits and on the actual use of publicly subsidized LTC.

The empirical literature on the determinants of LTC use suggests that there is an income gradient in formal LTC use in some European countries but not in others (Bakx, de Meijer, Schut, & van Doorslaer, 2015; Bonsang, 2009). Only four papers have specifically investigated horizontal inequity in LTC use by socioeconomic status. García‐Gómez, Hernández‐Quevedo, Jiménez‐Rubio, and Oliva‐Moreno (2015) find pro‐rich horizontal inequity among the elderly in Spain in 2008, that is, before public support for the disabled elderly was expanded. Exploiting the SHARE survey, Ilinca, Rodrigues, and Schmidt (2017) and Rodrigues, Ilinca, and Schmidt (2018) find that formal home care use is roughly proportionately distributed across income in most European countries. Also using SHARE, Carrieri, Di Novi, and Orso (2017) conclude that there is at most limited income‐related pro‐rich horizontal inequity in the use of nursing care and only in Southern Europe and in Nordic countries.

However, these studies are limited by data constraints in four ways. First, they do *not* include institutional care, which still represents the vast majority of LTC spending in OECD countries (e.g., two thirds in the Netherlands in 2012; Statistics Netherlands, 2012a, 2018a, 2018b, 2018c). Second, they only study whether one uses care, but ignore *how much* is being used, which may contribute to interpersonal variation in care use correlated with socioeconomic status. Third, income information obtained from surveys is subject to substantial reporting bias. Fourth, although SHARE is a large panel data set, the number of observations of elderly with functional limitations per country is limited. These drawbacks severely reduce the possibility to detect differences in LTC use across income. We overcome these data problems by using administrative data on the universe of LTC eligibility, LTC use, and income for all adults in the Netherlands.

Our paper also differs in the way horizontal inequity in care use is assessed. Distinguishing inequity from fair inequalities requires normative judgments about how to define needs and how different levels of care needs should result in different LTC uses, that is, what the norm of *vertical equity* in LTC use is (Fleurbaey & Schokkaert, 2011; Sutton, 2002; van Doorslaer et al., 2000). Usually, there is no directly observable measure of care needs. 2
In the health care system, the diagnosis and the provision of care are generally carried out by the same agent and through a decentralized process, at the level of care providers. Diagnoses then partly reflect providers' and system‐wide incentives to deliver a certain type and amount of care and are generally not recorded in a centralized way. Typically, studies of equity in care use derive an implicit norm of vertical equity in care use by regressing *actual* care use on the variables considered to lead to fair inequalities in care use (e.g., health status) and potential confounders (Wagstaff & van Doorslaer, 2000b). By contrast, we face a unique setting in which a measure of needs and an explicit norm for vertical equity are readily observable. We use the monetary value of the entitlements decided upon by the independent, central agency in charge of LTC eligibility assessments (called CIZ) as the sole indicator of legitimate needs for LTC. Furthermore, as we observe the ratio of average care use to average entitlements, we do not have to rely on the standard yet relatively strong assumption that there is no vertical inequity in LTC use on average. 3
We go one step further than Sutton (2002): he uses a data‐driven method to come up with a norm of vertical equity in LTC use, but still has to draw an arbitrary line between those individual characteristics considered to induce fair differences in LTC use and the non‐need factors. In our empirical analysis, this implies that the index of horizontal inequity we compute does not rely on any econometric estimate. Coupled with the exhaustive nature of our data, it results in a highly robust assessment of the discrepancy existing between the distribution of use and the distribution of entitlements across income.

We find LTC use to be more concentrated, in value, among the income‐poor individuals. Richer individuals are more likely not to use any care or to use home care services rather than (more costly) institutional care. These findings persist when differences in needs across income are controlled for. Interpreted literally, the marked income gradient in the need‐standardized LTC use suggests that the poor elderly receive “too much” LTC in comparison with richer elderly. Yet, the interpretation of the findings and their equity implications depend largely on the reasons why the allocation of LTC deviates from the distribution of entitlements.

## 2 THE DUTCH LONG‐TERM CARE SYSTEM

The Dutch public LTC insurance program (AWBZ) was started in 1968. 4
Between 2013 and 2015, the Dutch LTC system went through major changes (van Ginneken & Kroneman, 2015). We describe system as it stood in 2012, as we assess the pre‐reform situation. It offers universal and fairly comprehensive coverage. In 2014, 18% of the individuals aged 65 and older received public LTC support in the Netherlands (Muir, 2017; OECD, 2017). Private LTC is marginal. 5
Households' spending on non‐publicly subsidized long‐term care services was estimated to amount to 19 million euros in 2013. This represents less than 0.1% of the 24 billion euros of public and private spending on AWBZ‐financed LTC (Statistics Netherlands, 2017, 2018a, 2018b, 2018c). About 30% of public LTC beneficiaries aged 65 and older live in an institution, where they receive a package of services tailored to the type and severity of their disability (Table 1). At home, individuals may receive nursing care, personal care, individual, and group guidance. 6
Domestic help was delegated to municipalities in 2007 and is provided under a different scheme (Wmo).


**Table 1:** Types of LTC services

	Home care	Institutional care
Types of care	Nursing care, personal care, individual, or group guidance	Institutional stay
CIZ decision specifies:	Number of hours or half days of care and period of eligibility	Type of institution, “package” of services and period of eligibility

*Note.* LTC services financed by the public LTC insurance (AWBZ) in 2012.

Abbreviation: LTC, long‐term care.

To become eligible for publicly funded LTC, elderlies apply for eligibility at CIZ. Decisions regarding eligibility for publicly financed LTC are made by an assessor. These decisions are based on information that the assessor may gather about the functional limitations of the applicant, her or his health status, and a limited set of background characteristics as stated in a decision by the Minister of Health. This set does not include the applicant's income or wealth nor are assessors expected to take into account the size of the LTC budget in the applicant's region of residence. The presence of relatives reduces entitlements to LTC inasmuch as household members are capable of providing some minimum personal care to their disabled relative (Mot, 2010).

Assessors decide on the type volume of care and the length of time the applicant will be eligible for or whether the application is rejected altogether. When eligibility expires, there is a reassessment. Users may also request a reassessment when a change in their situation requires a different mix of LTC. Applicants may appeal decisions, but only 1% does so; 25% of appeals are approved (CIZ, 2014).

Beneficiaries can choose to receive in‐kind care, but they can also opt for LTC vouchers to pay their own professional caregivers or informal caregivers. Those with a more severe condition and a less supporting environment are made eligible for a nursing home admission; they may choose to stay home and receive home care or vouchers instead. 7
A more detailed description of the needs assessment process can be found in Bakx, Wouterse, van Doorslaer, & Wong (2018). he provision of in‐kind care is organized at a regional level: 32 regional purchasing agencies (*zorgkantoren*) are entrusted with this. In 2012, waiting lists for nursing homes and home care were short (CVZ, 2013).

Mandatory social security contributions and general government revenue pay for nine‐tenth of total costs (Schut et al., 2013); in 2012, only 8% was financed through cost‐sharing (Maarse & Jeurissen, 2016). 8
The agency in charge of computing the individual co‐payments, *Centraal Administratiekantoor*, is fully distinct from CIZ. Co‐payments increase with income, yet they do not exceed the cost of care nor the user's income. Furthermore, co‐payments are capped, with a monthly fee lower than €20 for beneficiaries with the lowest incomes. However, the co‐payments are higher for institutional care than for home care for individuals with a median or high income. In 2012, the monthly co‐payment reached €2,150 for a nursing home stay, but would not exceed €1,200 for home care.

Financial barriers in the access to LTC are thus limited in the Netherlands, especially for low‐income elderly. Yet, disparities in LTC use still might arise as a consequence of how LTC is delivered, for example, if some groups have better information about care options or if they receive priority. The Dutch Audit Office (Netherlands Court of Audit, 2015) and Duell, Lindeboom, Koolman, and Portrait (2019) documented that some variation in LTC use from one LTC purchasing region or CIZ regional office to the other cannot be explained by differences in entitlements. Whether those regional disparities or other features of the LTC system induce socioeconomic disparities in LTC use has not been documented to date.

## 3 EMPIRICAL STRATEGY

### 3.1 Measuring income‐related inequality in LTC use

We assess income‐related inequality in LTC use by computing the concentration index of LTC use, *CI*(*y*) (Wagstaff & van Doorslaer, 2000a). This index takes a value between −1 and 1; a negative (positive) concentration index indicates that, overall, there is some pro‐poor (pro‐rich) inequality in LTC use: Consumption is disproportionately concentrated among the less (more) well‐off. We express *CI*(*y*) as

(1) $$\mathrm{CI}\left(y\right)=\left(\frac{2}{\bar{y}}\right)\mathrm{cov}\left(y,r^{I}\right),$$

where *r*^*I*^ denotes the fractional rank in the income distribution of the population ( $r_{i}^{I}=i/N$ if *i* is the *i*‐poorest individual); *y* is a continuous and unbounded measure of LTC use, and $\bar{y}$ denotes the population average LTC use.

### 3.2 Controlling for differences in care entitlements

Not all income‐related inequality in LTC use should be considered as inequitable. In particular, heterogeneity in functional status may correlate with income and induce differences in LTC use along the income distribution that are considered to be fair. Finding out whether individuals with equal needs receive similar LTC requires an empirical measure of legitimate care needs.

In the Netherlands, entitlements for publicly subsidized LTC provide an explicit indicator of “eligible needs” in the public LTC insurance. Taking advantage of this unique feature of the Dutch LTC system and the fact that there is barely any privately paid LTC, we address the question: “How much of income‐related inequalities in the use of LTC services *cannot* be explained by differences in CIZ‐assessed needs (i.e., entitlements)?”

To compare the distribution of actual LTC *use* with the distribution of LTC *entitlements*, we define the horizontal inequity index of LTC use *HI*(*y*) as

(2) $$\mathrm{HI}\left(y\right)=\mathrm{CI}\left(y\right)-C^{N}\left(y\right),$$

where *C*^*N*^(*y*) stands for the concentration of entitlements (van Doorslaer & Van Ourti, 2011). In our context, we simply take it to equal the concentration *index* of entitlements, denoted *x*: *C*^*N*^(*y*) = CI(*x*). 9
We take *y* (respectively *x*) to be the monetary value of all LTC the individual used (respectively was entitled to) over year 2012 (cf. Section 4). We thus depart from the standard method, which consists of deriving the concentration of needs from a regression analysis of LTC use on LTC needs and potential confounding factors.

HI(*y*) can vary between −2 and +2. When positive (negative), it indicates that the rich (poor) receive more LTC services than the poor (rich), relative to their needs. 10
Inference on CI(*y*), CI(*x*) and HI(*y*) is described in Appendix S4. When the concentration of entitlements exactly mirrors the concentration of use across the income distribution, then there is no income‐related horizontal inequity and HI(*y*) = 0. Interpreting the magnitude of HI(*y*) is yet not straightforward; we therefore also derive *need‐standardized* LTC use for individual *i*, $y_{i}^{\text{IS}}$, based on an indirect standardization approach (O'Donnell, van Doorslaer, & Wagstaff, 2012). We define

(3) $$y_{i}^{\text{IS}}=y_{i}-\left(\frac{\bar{y}}{\bar{x}}\right)x_{i}+\bar{y},$$

where $\bar{x}$ is the population average of CIZ entitlements. The distribution of need‐standardized LTC use across income reflects the counterfactual concentration of LTC use if the differences in entitlements and income are uncorrelated. In line with this, there is a direct connection between need‐standardized use and the horizontal inequity index: $\mathrm{HI}\left(y\right)=\mathrm{CI}\left(y_{i}^{\text{IS}}\right)$.

Equation (3) makes clear that HI captures income‐related deviations of LTC use not directly from CIZ entitlements, but from $\left(\bar{y}/\bar{x}\right)x_{i}$. If we take CIZ eligibility decisions to be informative of the norm of equity in LTC use, then the LTC system would be both *horizontally* and *vertically* equitable if LTC use were equal to the entitlements for everyone. At the population level, the fact that LTC use is actually lower than entitlements (i.e., $\frac{\bar{y}}{\bar{x}}<1$; cf. Section 4.5) could be interpreted as evidence of *vertical* inequity. However, there can still be no *horizontal* inequity if actual use were equal to $\left(\bar{y}/\bar{x}\right)x_{i}$ for *all* individuals, that is, if the under‐use of care relative to entitlements were the same for all. 11
That the conversion of additional entitlements into actual use differs by income is one aspect of income‐related horizontal inequity in care use and is captured by HI (Fleurbaey & Schokkaert, 2011). We therefore capture the deviations from a distribution of use such that individuals with the *same* entitlements receive the *same* care (e.g., a situation with no horizontal inequity) but in which individuals with twice as much entitlements can receive less than twice as much care. In other words, our approach does not impose to rule out vertical inequity. 12
It may be questionable to label average under‐use of care as vertically *inequitable*, as it may be a result of personal preferences and not necessarily of external limitations (like waiting lists or lack of supply); but our approach does not require to take such a stance.


### 3.3 Decomposing inequality by sources and sub‐populations

In order to gain insights into the mechanisms behind income‐related inequalities in LTC use, we use the noncausal decomposition analysis presented in Wagstaff, van Doorslaer, and Watanabe (2003). The decomposition is based on an ordinary least squares regression of actual care use on care needs and other individual characteristics, as explained in supporting information Appendix S2. In addition, we replicate our baseline analysis on the subpopulation of the elderly eligible for home care on the one hand and on the subpopulation of those eligible for institutional care on the other hand.

## 4 DATA AND DESCRIPTIVE STATISTICS

### 4.1 Exhaustive administrative register data on LTC eligibility and use

We use a rich set of data sources covering the entire Dutch population that we link to detailed information on the eligibility decisions of CIZ that were valid in 2012; in particular, we know the reasons for which each individual has become eligible for LTC and the types and amounts of services he or she is entitled to receive. We link these data to information on the actual use of publicly subsidized LTC obtained from the Central Administration Office for public LTC insurance (*Centraal Administratiekantoor*). We further link tax records from 2011, which provide information on household income 13
Income includes labor income, pension benefits, income derived from assets, and government transfers. and assets, and data from the mandatory municipal registration (*Gemeentelijke Basisadministratie*) providing the LTC purchasing region the individual lives in, demographic characteristics (gender, age, marital status, and whether the individual has a foreign background) and household composition.

### 4.2 Population of interest

We focus on individuals aged 60 or older in 2012 and entitled to publicly subsidized LTC for a somatic or a psychogeriatric condition. Among those eligible only for institutional care in 2012, we exclude those who were eligible for a stay in a specialized institution other than a nursing home, a residential care home, a rehabilitation center, or a palliative care facility. 14
Other institutions include psychiatric hospitals and centers for the physically handicapped. We exclude individuals eligible for LTC due to mental health problems (other than psychogeriatric problems) or a physical or cognitive handicap: They have often lived for years with functional limitations and their use of LTC services and income situation may follow different patterns than those observed in the population affected by disability at an old age. We do not include individuals who were not eligible for public LTC *at any point* in 2012, that is, all individuals in our sample have nonzero LTC entitlements. 15
Given that LTC that is entirely privately financed is marginal in the Netherlands, virtually all elderly who are not eligible for publicly subsidized LTC have zero LTC use. Missing background information reduces sample size by less than 0.2%; the final sample includes 616,934 individuals.

### 4.3 Ranking variable

Individuals are ranked by their disposable income of year 2011, computed using the square root equivalence scale (OECD, 2011). 16
The income distribution is smooth; fewer than 500 individuals have an income equal to zero. We take income, rather than wealth, as the ranking variable because inequity in access to LTC is more likely to be related to income than to wealth in the Dutch context, for two reasons. First, there is a strong tie between income and the level of co‐payments for LTC, unlike for wealth in 2012. 17
Until 2013, only 4% of taxable wealth in excess of €21,000 per capita was added to the income sources taken into account to compute copayments (Non, 2017). Second, wealth differences in the Netherlands are rather small for two‐thirds of the population. Many Dutch households hold only a negligible amount of liquid assets (van Ooijen, Alessie, & Kalwij, 2014), 18
When social security wealth is not accounted for, as it is the case for the wealth information provided by tax records. Median wealth is of €38,000 in the sixth decile of the wealth distribution among the Dutch elderly, implying that 60% of the population have barely any wealth to spend on LTC (Statistics Netherlands, 2012b). possibly because of extensive public insurance against financial risks at old age. We yet assess whether *wealth‐related* horizontal inequity in LTC use goes in the same direction as income‐related inequity in a robustness check.

### 4.4 Measurement of LTC use and needs

The monetary value of annual LTC use is the sum of the value of in‐kind services used and of the imputed value of LTC vouchers. For in‐kind services, we multiply quantities used by their actual national tariff (for institutional care) or by the national price cap (for home care) set by the Dutch Healthcare Authority (NZA). If individuals opt for LTC vouchers rather than in‐kind care, we only observe whether they take up the vouchers, not the amount spent. We exploit the official matrix used to convert entitlements to in‐kind LTC into vouchers. 19
See Appendix S1. The cash equivalent of in‐kind services represents about 75% of their national price. LTC vouchers represented 9% of public spending on LTC in the Netherlands in 2013 (Statistics Netherlands, 2017, 2018a, 2018b, 2018c). On average, 89.5% of the value of vouchers granted is actually used (Statistics Netherlands, 2018b). We thus discount the imputed cash equivalent of entitlements to in‐kind services by 10.5% to obtain the individual imputed monetary value of vouchers being used. Similarly, entitlements are computed as the monetary value of eligible LTC services. 20
Eligibility for home care services is granted in hours per week and is expressed as a range (e.g., the individual can receive from 6 to 7 hr of nursing care per week); we take the middle point of the range (in our example, 6.5 hr) when computing the value of LTC the individual is eligible for, consistently with what is done when beneficiaries have their entitlements to in‐kind services converted into vouchers. Finally, we prorate the *annual* monetary values of LTC use and entitlements of individuals who died in 2012 using the proportion of the year they were alive. 21
For example, for an individual who died at the end of June, we multiply the value of her actual use of (needs for) LTC and her entitlements by two.


### 4.5 Descriptive statistics

Table 2 provides the summary statistics for the baseline sample. Almost two third of the study population was eligible for home care services, whereas less than half were eligible for institutional care, and about 12% of individuals were eligible for both types of care in year 2012 (Panel A). 22
An individual can be eligible for only one type of care at a point in time. However, she can have her needs reassessed and become eligible for another care setting. The average value of LTC an individual is eligible for is €31,000. The average value of entitlements for institutional care is higher than the average value of entitlements for home care because institutional care is generally more costly and individuals entitled to a nursing home stay have a worse functional status.

**Table 2:** Sample descriptive statistics (2012)

	Entire population		Eligible for	
			Home care	Institutional care
	Mean	*SD*	Mean	Mean
Panel A: Eligibility				
Eligible for home care (yes/no) (%)	65.0	—	100.0	25.4
Eligible for institutional care (yes/no) (%)	46.7	—	18.2	100.0
Value of entitlements to home care	12,179	25,686	18,726	4,052
Value of entitlements to institutional care	18,882	24,973	4,275	40,457
Value of total LTC entitlements (x)	31,061	29,871	23,000	44,509
Number of days with eligibility for LTC	255	132	243	280
Panel B: Use				
Any use of in‐kind home care (yes/no) (%)	61.5	—	85.4	36.4
Any use of institutional care (yes/no) (%)	38.7	—	13.4	82.9
Any take‐up of LTC vouchers (yes/no) (%)	4.4	—	5.8	2.4
Any use of LTC (yes/no) (%)	91.8	—	90.5	95.6
Value of in‐kind home care used	7,430	17,565	9,929	5,643
Value of institutional care used	14,595	23,580	2,216	31,271
Value of LTC vouchers used	935	6,572	1,161	823
Value of total LTC used (y)	22,960	26,664	13,307	37,737
Average value of use/average value of entitlements (%)	73.9	—	53.8	86.4
Panel C: Sociodemographic characteristics				
Gender: woman (%)	67.0	—	64.5	70.4
Age: 60–69 (%)	12.5	—	15.9	6.4
Age: 70–79 (%)	25.8	—	30.2	19.2
Age: 80–84 (%)	22.7	—	23.3	22.7
Age: 85–89 (%)	22.2	—	19.5	26.9
Age: 90+ (%)	16.8	—	11.1	24.7
Has died in 2012 (%)	16.0	—	13.1	21.1
Married/In a civil partnership (%)	34.5	—	40.4	26.2
Partner in household (%)	30.9	—	39.6	19.0
Number of household members	1.4	0.7	1.5	1.3
Origin: the Netherlands (%)	88.0	—	86.9	89.8
Origin: foreign Western country (%)	8.8	—	9.0	8.5
Origin: non‐Western foreign country (%)	3.2	—	4.1	1.8
Panel D: Economic resources				
Disposable income	25,117	16,259	25,659	24,309
Net wealth (per capita)	120,242	391,922	125,260	114,469
Owner of main residence (%)	32.2	46.7	36.7	26.3
Observations	616,934	—	401,262	287,932

*Note.* The population is composed of individuals 60 years and older who are eligible for LTC in the Netherlands in 2012 due to a somatic or psychogeriatric condition. Care use and care entitlement values are expressed in euros over the year 2012. Equivalized income (over the year 2011) is expressed in euros. Wealth (as of December 31, 2011) is expressed in euros.

Abbreviations: LTC, long‐term care; *SD*, standard deviation.

As shown in Panel B, the average value of the used LTC is €22,960. Seventy percent of individuals use less LTC than they are eligible for, and about 8% did not use any in‐kind care or vouchers at all. This might be because they apply for eligibility for LTC out of precaution or because the elderly and their families think the marginal costs outweigh the marginal benefits. Also, the average care use is found to be only three fourth (73.9%) of the average value of the care patients were eligible for. In the subpopulation eligible for home care, only half of the care entitlement is used. 23
The finding that average long‐term care use is substantially lower than average entitlements justifies why we do not take a situation in which long‐term care use equals entitlements for everyone as the reference point (cf. Equation 3).


Most individuals in the study population are women and in their 80s (Panel C). Seventy percent of the sample lived without a partner for most of 2012; only 7% of them spent more than half of the year in a nursing home.

## 5 RESULTS

### 5.1 Differential care use for equal needs

**Figure 1:**
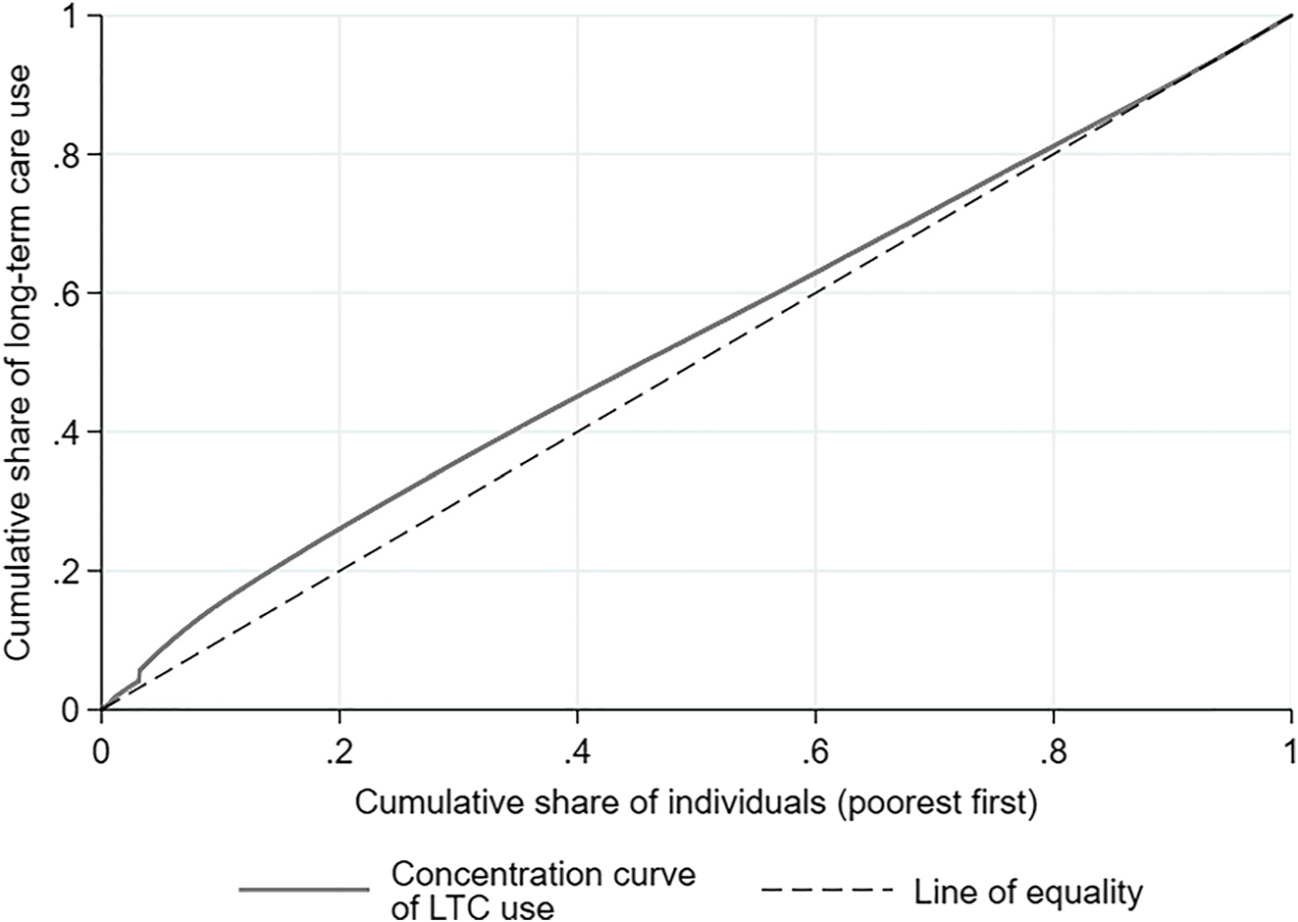
Concentration curve of LTC use (*y*). The population is composed of individuals 60 years and older who are eligible for public LTC in the Netherlands in 2012 due to a somatic or a psychogeriatric condition (*N* = 616,934). LTC use is expressed in annual monetary value. Individuals are ranked by their 2011 disposable income. Abbreviation: LTC, long‐term care

As shown in Table 3, the concentration index of LTC use is negative, reflecting a pro‐poor concentration of LTC use. The concentration curve of LTC use (see Wagstaff & van Doorslaer, 2000a) is above the line of equality almost over the entire income distribution (Figure 1): This means that, for instance, the 30% poorest individuals consumed 36.0% (i.e., more than 30%) of the total value of LTC services used in 2012.

**Table 3:** Concentration and horizontal inequity indexes of long‐term care use

	*CI*(*y*)	*CI*(*x*)	*HI*(*y*)	*N*
	(1)	(2)	(3)	
Entire sample	−0.0596*** (0.0008)	−0.0254*** (0.0007)	−0.0342*** (0.0004)	616,934
Eligible for home care	−0.0239*** (0.0018)	0.0027* (0.0015)	−0.0267*** (0.0011)	401,262
Eligible for institutional care	−0.0392*** (0.0008)	−0.0179*** (0.0006)	−0.0213*** (0.0004)	287,932

*Note.* The population is composed of individuals 60 years and older who are eligible for home care in the Netherlands in 2012 due to a somatic or a psychogeriatric condition. Standard errors in parentheses.

* *p*<.10.

** *p*<.05.

*** *p*<.01.

However, poorer elderly also have higher assessed needs: *CI*(*x*) is negative (−0.0254). The pro‐poor concentration of needs is yet lower than the pro‐poor concentration of actual use; this results in a negative horizontal inequity index (of −0.0342), which implies that, even when correcting for differences in entitlements, the poor receive more LTC (in value) than the rich.

**Figure 2:**
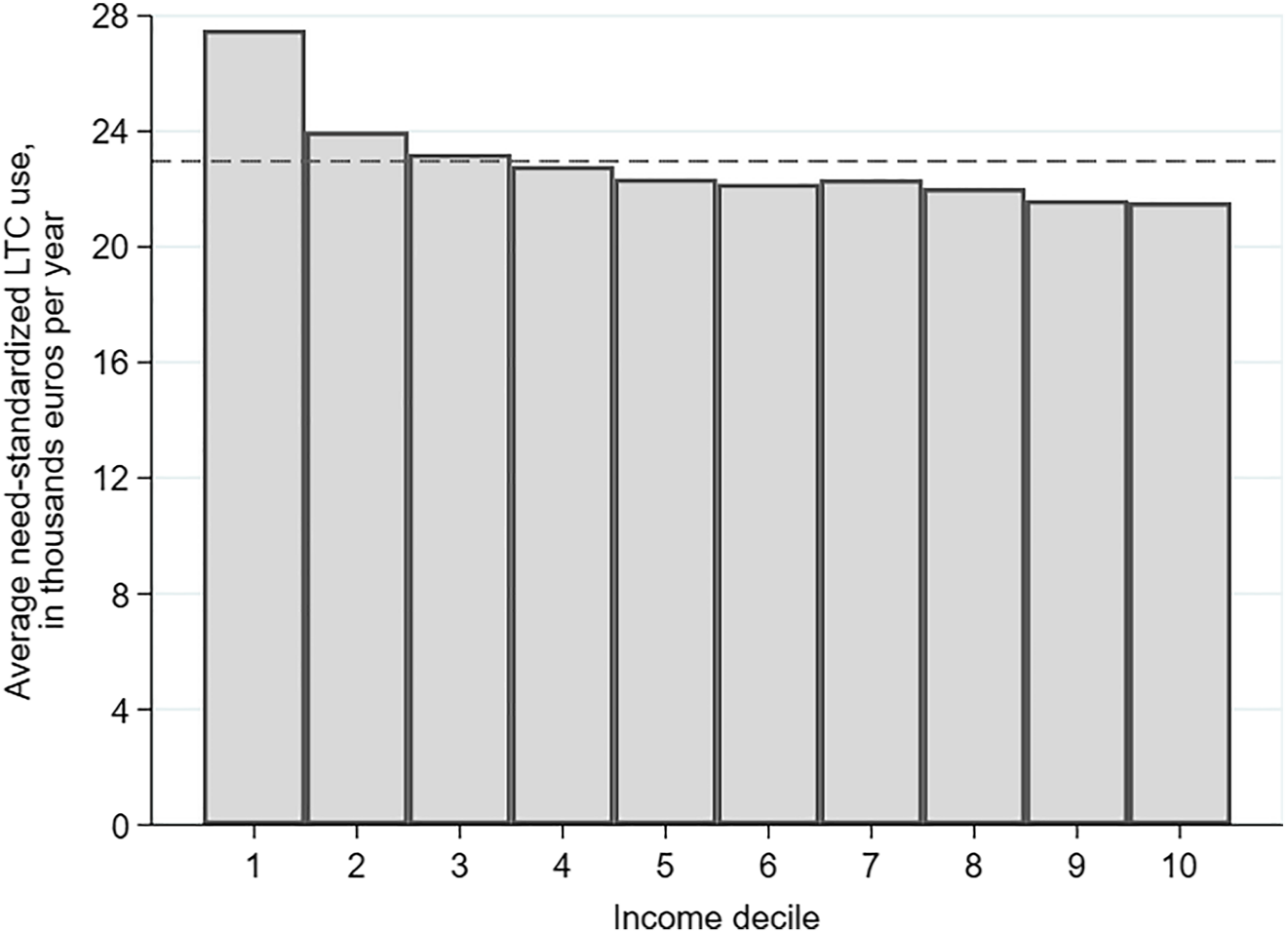
Average need‐standardized LTC use (*y*^*IS*^) by income decile. The population is composed of individuals 60 years and older who are eligible for public LTC in the Netherlands in 2012 due to a somatic or a psychogeriatric condition (*N* = 616,934). Need‐standardized LTC use is expressed in annual monetary value. The dashed horizontal line indicates the population‐average value of LTC use. Abbreviation: LTC, long‐term care

Figure 2 displays the average need‐standardized LTC use per income decile, and it highlights two findings. First, the differences are sizable: After need standardization, horizontal equity would require that all deciles use the same amount of care, but the 10% poorest elderly are predicted to use 28% more LTC than the 10% richest. Second, the difference between income deciles is larger at the bottom of the income distribution.

### 5.2 Decomposition of the horizontal inequity index

**Figure 3:**
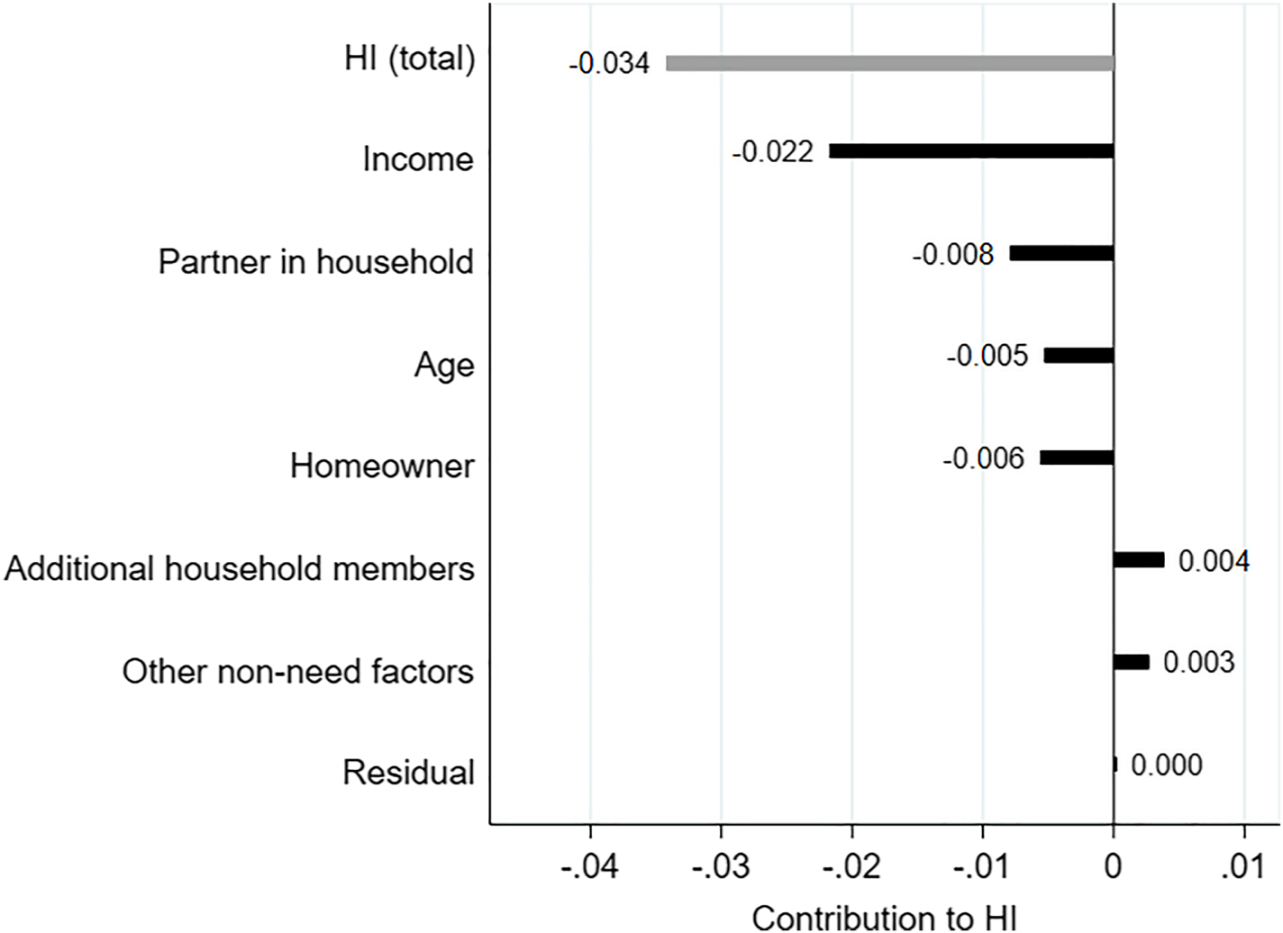
Decomposition of the horizontal inequity index of long‐term care (LTC) use (*HI*(*y*)). The population is composed of individuals 60 years and older eligible for public LTC in the Netherlands in 2012 due to a somatic or a psychogeriatric condition (*N* = 616,934). Variables depicted on the right‐hand side of 0 contribute to pro‐rich inequality in LTC use; variables depicted on the left‐hand side of 0 would contribute to pro‐poor inequality. Gender, wealth, and region of residence are grouped in the category “Other non‐need factors” as the contribution of each of these factors is lower than 0.003 in absolute value. Reading: income, for example, contributes to pro‐poor inequality by −0.022 (out of a total horizontal inequity index of −0.034) [Colour figure can be viewed at http://wileyonlinelibrary.com]

What factors are associated with the discrepancy between entitlements and use? To answer this question, we decompose HI into the contributions of non‐need factors. 24
The results from the ordinary least squares regression as well as the concentration indexes of non‐need factors are presented in Appendix S2. We include age (in five brackets), gender, whether the individual has a partner living in the house, the number of household members, the migrant background of the individual or of her parents (seven origins), home ownership, income decile, wealth decile, and a dummy for the LTC purchasing region the individual lives in. Results are shown in Figure 3. Characteristics can only contribute to HI when they correlate with income (i.e., have a non‐zero regression coefficient) and are unequally distributed across income (i.e., show a non‐zero concentration index).

We do find evidence of regional disparities in LTC use even when entitlements and the socioeconomic and demographic composition of the disabled elderly population are taken into account. 25
Duell, Koolman, and Portrait (2017) find that practice variation in *eligibility* for nursing home care across the 10 CIZ regional offices is limited. Our results show that looking at how actual care use varies across the 32 LTC purchasing regions, for given entitlements, reveals substantial disparities. We can only speculate about the origins of such inequalities: They may come from, among other things, disparities in the supply or regional differences in the willingness or ability to substitute informal care for formal care. What our decomposition does reveal, however, is that it is *not* the case that regions with relatively higher use are on average poorer than “under‐consuming” regions. 26
This result complements the findings from a recent paper by Duell et al. (2019) assessing practice variation in nursing home care in the Netherlands. The authors first calculate the difference between observed use and use predicted based on applicants' case mix; subsequently, they test if this difference varies across CIZ regional offices. As the variation is the same across all income quintiles, the authors conclude that income does not contribute significantly to practice variation in nursing home access. In other words, the LTC purchasing region in which individuals live does not contribute to the income‐related HI index.

By contrast, age *does* contribute slightly to the disproportionate LTC use of low‐income elderly: Being older is associated with higher use, even for given needs, and the eldest also tend to be poorer. Also, home owners tend to be richer and are less likely to use residential care.

### 5.3 Inequity in home care and institutional care use

Although our baseline analysis provides an overall picture of inequalities in LTC use, it combines two very different populations: individuals eligible for institutional care and those eligible for home care. We replicate the analysis for these two (partly overlapping) subgroups. LTC use is equal to all LTC consumed *while* the individual was eligible for either home care or institutional care. Individuals eligible for institutional care are on average older, more often female, single and with no migrant background, and have lower wealth and income than those eligible for home care (Table 2).

**Figure 4:**
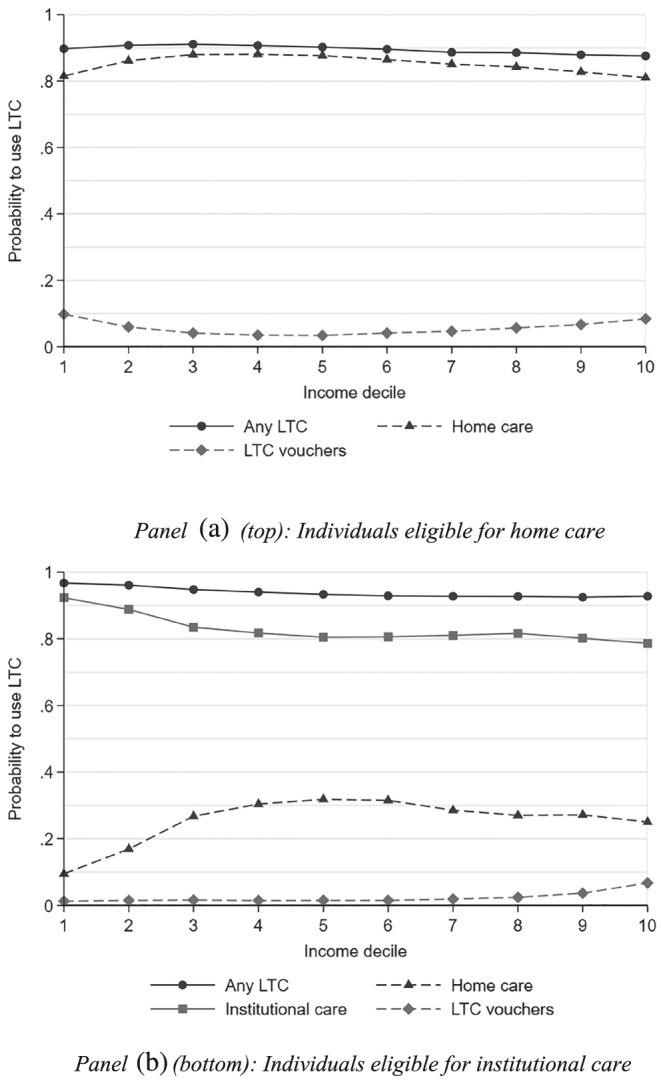
Probability of using a given type of LTC, by subgroup. The population is composed of individuals 60 years and older eligible for public home care (Panel A) or institutional care (Panel B) in the Netherlands in 2012 due to a somatic or a psychogeriatric condition (*N* = 401,262 in Panel A; *N* = 287,932 in Panel B). A given individual may use several types of care over the period he or she is entitled to receive home care or institutional care. The probabilities are not adjusted for care needs. Abbreviation: LTC, long‐term care

We observe six main patterns. First, in both subgroups, the pattern of LTC use changes with income (Figure 4). Among the elderly eligible for institutional care, the probability to use some institutional care decreases from around 90% in the two bottom income deciles to about 80% for the 70% richest individuals (Panel B); among those eligible for home care, the probability to take up LTC vouchers rather than in‐kind care is highest at both the bottom and the top of the income distribution (Panel A).

Second, these patterns persist when we control for differences in needs across the income distribution in each subgroup. In particular, a €1 increase entitlements for home care results in €0.53 additional LTC use for individuals in the bottom half of the income distribution *versus* €0.47 in the top half of the income distribution. 27
Among the elderly eligible for institutional care, €1 more of entitlements results in €1.012 of additional LTC use for the 50% poorest individuals and €0.996 more for the top half. Given the high average value of LTC needs for the elderly eligible for institutional care (k€40), this small gap translates into sizable differences in average LTC use across income for given needs. Consistently, HI(*y*) is negative for both types of LTC (Table 3).

Third, CI(*x*) is close to zero in the subgroup eligible for home care: Unlike in the population eligible for any LTC, we do not find lower income to be associated with higher entitlements. This may be because higher income individuals are better able to postpone a nursing home admission (*selection effect*), for example, because they are more likely to have a house that is fit for aging in place or that may be adapted more easily. Furthermore, income differences by gender and household composition—women and singles are more often poor—also contribute to the greater needs for community‐dwelling individuals in upper income deciles (*composition effect*). 28
Among the elderly eligible for home care, women have on average 12% lower LTC entitlements, and there are relatively more females in the bottom of the income distribution; those living with a partner have on average 7% higher entitlements than singles, and the proportion of couples increases with disposable income. Finally, a zero or positive CI(*x*) may reflect income‐related differences in the ability to navigate the LTC system and in the propensity to claim certain LTC services. 29
When claiming LTC, individuals may also specify which types of care they would like to receive; a stronger preference for home care over institutional care among higher income elderly could then explain our finding. Yet documentation about the assessment procedure shows that the preferences expressed by applicants need not be taken into account. According to Bakx et al. (2018) who interviewed several CIZ assessors, these preferences rarely play a role in the assessment process.


**Figure 5:**
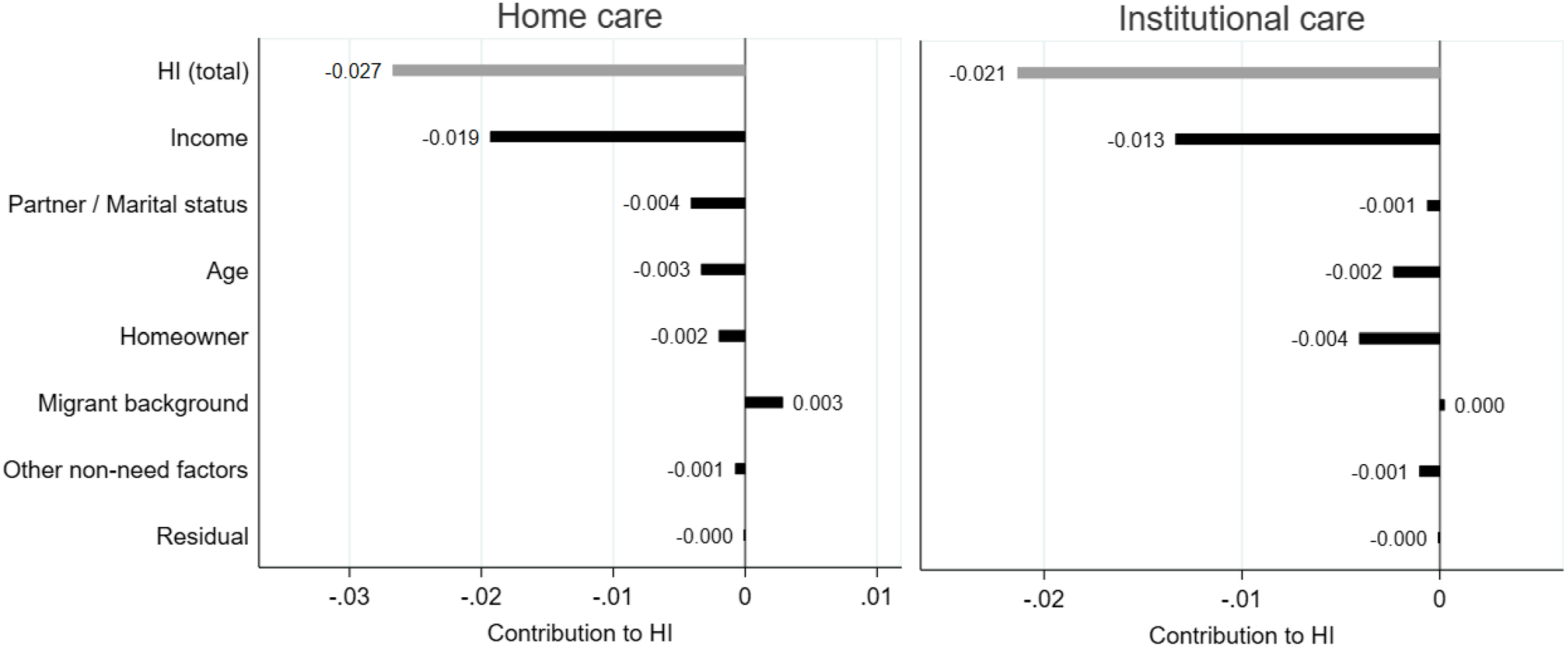
Decomposition of the horizontal inequity index of long‐term care use (*HI*(*y*)), by subpopulation. The population is composed of individuals 60 years and older eligible for public home care (*N* = 401,262) or institutional care (*N* = 287,932) in the Netherlands in 2012 due to a somatic or a psychogeriatric condition. When focusing on institutional care, the non‐need factors “having one's partner in own household” is replaced by marital status, and the contribution of “household size” is not estimated. Gender, wealth, household size (if applicable), and region of residence are grouped in the category “Other non‐need factors” as the contribution of each of these factors is lower than 0.002 in absolute value in both subgroup analyses

Fourth, when decomposing HI(*y*), the contribution of income is large in both subgroups, relative to the contributions of other factors (Figure 5). That income per se is strongly associated with lower LTC use may reflect the impact of co‐payments, which are higher for higher income individuals. In particular, as the (absolute) difference in co‐payments between institutional care and home care increases with income, higher income elderly have a stronger financial incentive to use home care or vouchers than the poor. 30
Evidence on the price elasticity of institutional care use is mostly grounded in the U.S. context. Some papers have found the elderly do not adjust their use of nursing home care to its out‐of‐pocket price (e.g., Grabowski & Gruber, 2007), whereas others have found nonzero price elasticities (e.g., Reschovsky, 1998; Hackmann & Pohl, 2018). Home care use has been shown to be moderately price elastic in Europe (Roquebert & Tenand, 2017). Besides concerns about the validity of the available estimates in the Dutch context, the lack of evidence on the cross‐price elasticity of home care and institutional care as well as the complexity of the Dutch co‐payment schedule make it difficult if not impossible to predict the extent to which co‐payments may account for the pro‐poor concentration of LTC use that we observe. But the large contribution of income might also be driven by unobserved determinants of LTC use correlated with income, such as the accessibility or adaptation of the house (Diepstraten, Douven, & Wouterse, 2019).

Fifth, gender differences contribute to the pro‐poor inequity in home care use: Women—who rank on average lower in the income distribution than their male counterparts—use about 14% more (formal) home care than men after standardizing for needs. Women are found to receive less informal care from their spouse than men in several countries (Katz, Kabeto, & Langa, 2000); the difference in this population is primarily due to women being more often single than men, and having a partner in the house being associated with lower LTC use. The gender gap in need‐standardized home care use is similar among singles and the elderly living with a partner (*result not shown*).

Finally, the elderly with a migrant background other than those coming from a Western country or a former Dutch colony are found to have substantially lower need‐standardized home care use, while being on average poorer.

### 5.4 Robustness of the results

We perform two robustness checks. First, we study the impact of how we deal with LTC users who died during 2012 (16% of the population). As mortality rates are not equal across income groups, excluding these individuals from our analysis may bias our assessment of income‐related inequalities in LTC use. On the other hand, pro‐rating the LTC needs and use of those who have died in the year creates some outliers with respect to our two main variables of interest. We have therefore checked that the concentration and horizontal inequity indexes remain similar when we keep only individuals who have survived throughout the year (Table S3.1 of Appendix S3.1).

Second, we check that the potential unobserved heterogeneity in the utilization rate of LTC vouchers (assumed to be 89.5% across all income levels) does not drive the pro‐poor concentration of LTC use. Even under the extreme assumption that the elderly in the top decile use 100% of their vouchers and the bottom decile use 0%, the gap in LTC use (after standardization for entitlements) would still be of 22%, compared with 28% in our baseline analysis. The take‐up rate of vouchers (Table S5.2 of Appendix S5) is too small for differences in the utilization rate of vouchers to significantly affect HI estimates.

As a complementary analysis, we use per capita household wealth as reported to the tax office rather than income as the ranking variable. Differences in need‐standardized LTC use across wealth deciles are limited (Appendix S3.2). Although individuals in the bottom wealth decile tend to use *less* LTC than richer individuals when eligible for home care, 31
Rodrigues et al. (2018) show that using wealth rather than income as the ranking variable results in a more pro‐poor distribution of home care use when controlling for (statistically derived) needs, in most European countries—except for the Netherlands and Belgium. among those eligible for institutional care, we find that the elderly in the top three wealth deciles use slightly less LTC, for given entitlements.

## 6 DISCUSSION

We have tested whether the Dutch elderly receive equal care for equal needs by using their entitlements to publicly subsidized LTC as a measure of care needs. Six key results emerge. First, rich or poor, most elderly use less publicly subsidized LTC than what they are entitled to. This cannot be explained by a shortage of LTC supply, as none was observed in 2012. Second, under‐use compared with care entitlements is more pronounced among the rich than among the poor. Thus, the concentration of LTC use is pro‐poor even after we control for differences in entitlements by income. Third, we find a pro‐poor HI index both in the subpopulation eligible for home care and in the subpopulation eligible for institutional care, even though CIZ‐assessed needs do not appear to be higher when income is lower among the elderly eligible for home care, possibly due to composition and selection effects. Fourth, among the elderly eligible for institutional care, richer individuals are more likely to forgo LTC or to use home care instead. Fifth, income per se has a strong negative association with LTC use when other non‐need factors are controlled for. Finally, regional disparities in need‐standardized LTC use cannot be explained by regional differences in the sociodemographic composition of the disabled elderly population, nor by “under‐consuming” regions being systematically richer or poorer than the other regions.

What do our results imply for the performance of the Dutch LTC system and its capacity to ensure horizontal equity in LTC use?

The finding that income‐related disparities in LTC use do not fully mirror income‐related disparities in entitlements seems to suggest that the Dutch system falls short of its egalitarian objective in the provision of LTC services and results in horizontal inequity in LTC use favoring the low‐income elderly. However, this interpretation hinges upon three conditions. These three conditions are not specific to our study setting only; they apply to the interpretation of most of the literature on equity in access to health care, including most seminal papers cited in Wagstaff et al. (2000) and van Doorslaer and Van Ourti (2011). However, they are often taken for granted in the context of access to medical care. We revisit them here because they aid to place the findings into perspective and suggest directions for further research.

First, CIZ entitlements have to be a relevant and unbiased indicator of LTC needs. We hypothesize that CIZ eligibility decisions are informative of the policy objectives regarding access to LTC in the Netherlands because the guidelines for needs assessments are derived from a Ministerial decision on eligibility and CIZ is an independent and centralized organization exclusively in charge of conducting needs assessments (RMO, 2010; Schut & van den Berg, 2010). But if the well‐off are better capable of navigating the LTC system and more likely to claim that they need care (e.g., out of precaution), relying on CIZ‐assessed needs may hide socioeconomic inequity at the stage of the eligibility decision.

Second, it has to be relevant to focus on inequalities in public *formal* LTC use, independently from the allocation of private LTC and informal care. The choice of the outcome depends on whether public LTC is provided independently from informal care and private LTC options or merely intended as a safety net for when these other sources are not available. Societies may hold different viewpoints on this. In the Netherlands, public financing of formal LTC is comprehensive, yet the needs assessment process explicitly factors in the expectation that household members will provide some “usual care.” Whether the elderly have access to formal LTC independently from the informal care they may receive *beyond usual informal care*, thus is the relevant metric in this context, and it is the one we use in our analysis.

The third condition for our results to indicate that the Dutch LTC system unduly favors lower income elderly is that a lower use of institutional care is considered a disadvantage. If differences in the propensity to age in place across the income distribution stem from *differences in preferences* over care arrangements, income‐related disparities in need‐standardized LTC use may not necessarily be unfair. 32
Respect for preferences is a feature of responsibility‐sensitive egalitarianism (Fleurbaey & Schokkaert, 2011). However, co‐payments for LTC are up to 100 times higher for the income‐rich than the poor, and the difference is larger for nursing home stays than for home care; we speculate that such a schedule might contribute to the better‐off elderly being more likely than low‐income elderly to substitute home care or LTC vouchers for institutional care. Furthermore, if home‐based LTC were favored over institutional care, *irrespective of one's socioeconomic status*, then our results reflect that lower income elderly do not have effective access to most‐valued care options. Then, the income gradient in institutional care use would actually reflect the limited opportunities of low‐income elderly to age in place.

Our cross‐sectional study does not show the impact of income‐related disparities in life expectancy and time lived disabled on equity in LTC use. Future research using longitudinal data may be able to show how socioeconomic inequity in access to LTC may rise or fall over the life cycle.

Furthermore, we only focus on equity in access to LTC, in isolation from other determinants of well‐being in old age. Although partial equity assessments have been challenged on conceptual grounds (Fleurbaey & Schokkaert, 2011), we have three reasons for this choice. First, in 2012, the Dutch LTC social insurance was set apart from other health care; so substitution between LTC services and health care is expected to be low, although some substitution between home care and locally provided, tax‐funded domestic help seems to be taking place (Kattenberg & Bakx, 2018). Second, policy makers and the public opinion have voiced concerns relating to LTC access independently from other care and assistance services; the right to affordable LTC is also recognized as a distinct social right by the European Union (European Commission, 2019). Third, we believe that our analysis offers more policy guidance if it focuses on a well‐identified policy domain (elderly care).

## 7 CONCLUSION

On the whole, our findings do not offer unambiguous support for the common view that universal access and generous coverage in the Dutch public LTC insurance results in those with equal entitlements receiving similar LTC. On the one hand, the “Dutch model” ensures that poorer elderly do not forego a nursing home stay or intensive home care for financial reasons; on the other hand, the less well‐off may still lack sufficient support to age in place, whereas financial incentives may inefficiently keep some richer elderly out of a nursing home. One lesson to be drawn for other countries is that expanding public LTC insurance will not necessarily crowd out disparities in access.

From a methodological viewpoint, our analysis is unique in that we do not rely on any arbitrary assumption nor econometric analysis to adjust the distribution of LTC use for the distribution of entitlements. It paves the way for similar studies on the allocation of LTC in countries where administrative data on the entitlements to publicly subsidized LTC are available.

Our analysis focuses on 2012. Between 2013 and 2015, the Dutch social LTC insurance was substantially reformed: Criteria to be admitted to a nursing home were tightened, the differentiation by wealth in the level of public co‐payments has been increased (and subsequently reduced in 2017), and certain home care services have been transferred to municipalities whereas the provision of personal and nursing care is now the responsibility of regional health care providers (Maarse & Jeurissen, 2016). The effects on equity in LTC access have been vividly debated in the Netherlands; in order to later assess the impact of the reforms, it was essential to map the pre‐2013 situation with respect to equity.

## FUNDING INFORMATION

Access to the data was funded by Labex OSE ‐ *Ouvrons la Science Economique* and by the Network for Studies on Pensions, Aging and Retirement (NETSPAR) within the project “Optimal saving and insurance for old age: the role of public long‐term care insurance.” M. Tenand acknowledges support from Fondation Médéric Alzheimer to her doctoral research.

## ACCESS TO AND USE OF NDIVIDUAL‐LEVEL DATA

The results presented in this article are based on calculations by the authors using nonpublic microdata from Statistics Netherlands (CBS). Under certain conditions and a confidentiality agreement, these microdata are accessible for statistical and scientific research. For further information: microdata@cbs.nl. Exploitation of the data and publication of the results are made in compliance with the European privacy legislation (GDPR, May 25, 2018).

## CONFLICT OF INTEREST

M. Tenand reports personal fees from Fédération française des assurances (FFA), for a consultancy mission outside the submitted work. P. Bakx reports grants from the National Institute for Public Health and The Environment and CPB Netherlands Bureau for Economic Policy Analysis outside the submitted work and he uses data from the Centrum Indicatiestelling Zorg (CIZ) for other research. E. van Doorslaer has nothing to disclose.

## Supporting information

Data S1: Supporting Information
