# Supplementary material for: Equal long‐term care for equal needs with universal and comprehensive coverage? An assessment using Dutch administrative data
Source: Health Econ. 2020 Jan 20;29(4):435–51. doi: 10.1002/hec.3994 (PMC7078855; doi:10.1002/hec.3994)
Supplement: Supplementary file 1 — Data S1: Supporting Information [file HEC-29-435-s001.pdf]

Supplementary material

to the article

Equal long-term care for equal needs with universal and comprehensive coverage? An assessment using Dutch administrative data

September, 20th 2019

Contents

A National tariffs and price caps in the Dutch long-term care public insurance 2

B Deriving and decomposing the Horizontal Inequity index 4

B.1 Deriv the Horizontal Inequity index 4

B.2 Decomposition of Horizontal Inequity index 4

B.3 Linear estimates 6

B.4 Concentration indices of CIZ-assessed needs and non-need factors 13

C Robustness 14

C.1 Excluding individuals who died in 2012 14

C.2 Wealth-related inequalities in LTC use 15

D Inference 17

E Additional descriptive statistics 19

E.1 Income, wealth, LTC entitlements and use 19

E.2 Take-up of LTC vouchers by income deciles 19

A National tariffs and price caps in the Dutch long-term care public insurance

The monetary costs of LTC services funded through the public insurance system (AWBZ)[1] are computed using a national grid of tariffs, presented in Table A.1.

LTC institutions (nursing homes and residential care homes), which are public in the Netherlands, receive funding in accordance with this grid. Providers of home care services are mostly private; those covered by the public LTC insurance contract with the regional authorities and agree on hourly prices that should not exceed the national tariffs reported below.

Table A.1: Tariffs of LTC services by the Dutch public LTC insurance (AWBZ) in 2012

Home care services		Nursing and residential care homes	
Type of care	Tariff/hour	Level of services (ZZP package)	Tariff/day
Personal care	€49.81	Level 1	€63.03
Guidance	€57.75	Level 2	€80.44
Nursing care	€71.52	Level 3	€98.07
		Level 4	€113.117
		Level 5	€174.32
		Level 6	€168.28
		Level 7	€210.35
		Level 8	€239.14
		Level 9	€211.21
		Level 10	€259.72

SOURCES: Nederlandse Zorgautoriteit (2011b,a).
NOTES: In the Netherlands, domestic help is not funded by the public LTC insurance. The ZZP packages we refer to correspond to institutional care of type *Verpleging en verzorging* (stays in a nursing home, residential care home, rehabilitation center or palliative care center).

When individuals are eligible for institutional care but they choose to stay in the community and receive home care services instead, the package of services they would have received in an institutional setting is converted into a certain package of home care services, following the grid presented in Table A.2.

Table A.2 also provides a comparison between the monetary value of an institutional stay and the monetary value of the equivalent package of home care services. The absolute difference between monetary values, as a function of the level of services (thus, roughly as a function of the severity of disability), is U-shaped. The difference exceeds a hundred euros per week for low levels of disability; it decreases until the two living arrangements are equally costly (for ZZP package 7), before increasing again for the most severe disability levels. Individuals who opt for home care when they are eligible for institutional care

[1]We refer to the LTC system as of 2012.

with low and high levels of LTC services have thus a lower value of LTC use than similar individuals opting for a stay in an institution.

Table A.2: Correspondence between institutional care and home care: Official conversion grid and comparison of costs

	Hours of home care services, per week			Monetary value, per week		
	Personal care	Nursing care	Guidance	Cost of home care equivalent	Difference institutional care − home care	Ratio of home care cost /institutional care cost
Level of services (ZZP package)	(1)	(2)	(3)	(a)	(b)	(c)
Level 1	1	1.5	3	€330	€110	74.9%
Level 2	5.5	1.5	1	€440	€123	78.1%
Level 3	8.5	1.5	1	€589	€97	85.8%
Level 4	5.5	1.5	5.5	€699	€92	88.3%
Level 5	5.5	5.5	8.5	€1,158	€61	95.0%
Level 6	8.5	5.5	5.5	€1,135	€42	96.4%
Level 7	8.5	5.5	11.5	€1,481	-€9	100.6%
Level 8	11.5	5.5	11.5	€1,631	€42	97.5%
Level 9	8.5	5.5	8.5	€1,308	€170	88.5%
Level 10	14.5	8.5	5.5	€1,649	€168	90.7%

SOURCE: College voor Zorgverzekeringen (2012); Nederlandse Zorgautoriteit (2011b,a). Authors' computations of weekly monetary values.
READING: An individual made eligible for institutional care with a ZZP package of level 1 will be equivalently entitled to receive 1 hour of personal care, 1.5 hour of nursing care and 3 hours of guidance per week. The monetary value of home care services equivalent to the level of services the individual would receive in institution represents 74.9% of the monetary value of the institutional care she is entitled to.
NOTES: The ZZP packages we refer to correspond to institutional care of type *Verpleging en verzorging* (stays in a nursing home, residential care home, rehabilitation center or palliative care center).

Individuals eligible for either home or institutional care can also opt for LTC vouchers (or a combination of vouchers and in-kind care). If the individual is eligible for institutional care, her entitlements are first converted into a package of home care services (grid in Table A.2). The value of the LTC vouchers is equal to the monetary value of the package of home care services, computed using the tariffs of Table A.1, minus a 25% discount.

B Deriving and decomposing the Horizontal Inequity index

B.1 Deriv the Horizontal Inequity index

The Horizontal Inequity (HI) index has been developed as a measure of relative (income-related) horizontal inequity. Contrary to previous studies, which had to rely on a regression-based derivation of needs, our data and the institutional context allow us to observe a measure of needs - the entitlements of LTC, x_i.

HI for LTC use (y_i) is then simply derived as the difference between the concentration index of LTC use and the concentration index of LTC entitlements:

$$HI(y) = CI(y) - CI(x) \tag{1}$$

As soon as all income groups consume the same proportion $0 < \delta \leq 1$ of their LTC entitlements (thus $CI(y) = CI(\delta x) = CI(x)$), there is no relative income-related horizontal inequity: $HI(y) = 0$.

B.2 Decomposition of Horizontal Inequity index

The standard decomposition formula

We refer to a decomposition technique to highlight the individual characteristics that correlate most strongly with both income and LTC use, as proposed by Wagstaff et al. (2003). Assume that LTC use depends on care needs (here entitlements x_i) and a set of K 'non-need factors' z_i^k in the following way:

$$y_i = \beta_0 + \beta^N x_i + \sum_{k=1}^{K} \beta_k^{NN} z_i^k + \epsilon_i \tag{2}$$

with ϵ being an error term.

Referring to the parameters from Equation (2), income-related inequality in LTC use can be decomposed as (Wagstaff et al., 2003; O'Donnell et al., 2012):

$$CI(y) = \beta^N \frac{\bar{x}}{\bar{y}} CI(x) + \sum_{k=1}^{K} \Big[(\beta_k^{NN} \frac{\bar{z}^k}{\bar{y}}) CI(z^k) \Big] + \frac{2cov(\epsilon, r^I)}{\bar{y}} \tag{3}$$

$$= C^N(y) + C^{NN}(y) + \frac{2cov(\epsilon, r^I)}{\bar{y}} \tag{4}$$

$$= C^N(y) + HI(y) \tag{5}$$

where r^I the fractional rank in the income distribution. $\bar{y}$ (respectively $\bar{x}$ and $\bar{z}^k$) denotes the population-average of variable y (resp. of x and z^k). $C^N(y)$ denotes the concentration of care needs. $C^{NN}(y)$ represents the total contribution of the observable non-need factors of care to the concentration index of LTC use; $2cov(\epsilon, r^I)/\bar{y}$ is the generalized concentration index of the error term and captures the degree of correlation between LTC use and the income rank that is not explained by neither CIZ-assessed needs nor non-need factors.

Implementation in our study

In order to ensure the consistency of formulas (2) and (3), we estimate a constrained version of Equation (2):

$$y_i = \frac{\bar{y}}{\bar{x}} x_i + \sum_{k=1}^{K} \beta_k^{NN} z_i^k + \epsilon_i \tag{6}$$

meaning that we impose: (1) $\beta^N = \bar{y}/\bar{x}$ and (2) $\beta_0 = 0$.

$CI(y)$ can thus be decomposed as:

$$CI(y) = \frac{\bar{y}}{\bar{x}} \frac{\bar{x}}{\bar{y}} CI(x) + \sum_{k=1}^{K} \Big[\big(\beta_k^{NN} \frac{\bar{z}^k}{\bar{y}}\big) CI(z^k) \Big] + \frac{2cov(\epsilon, r^I)}{\bar{y}} \tag{7}$$

$$= CI(x) + HI(y) \tag{8}$$

Assumption (1) ensures that the concentration of needs is exactly measured by the concentration index of CIZ-assessed needs ($C^N(y) = CI(x)$); consistent with Equation (1), $HI(y)$ captures the discrepancy between the concentration of use and the concentration of entitlements.

From a theoretical perspective, it allows us to capture horizontal inequity in use as the deviation from a situation in which all individuals with the same needs have the same LTC use ("equal care for equal needs"), but in which average LTC use can possibly differ from average entitlements. Remember that average LTC use value represents less than 3/4 of average entitlements in our study population; acknowledging that $\bar{x} > \bar{y}$, horizontal equity in LTC use only requires that everyone uses the same fraction $\bar{y}/\bar{x}$ of her or his entitlements.[2] In particular, *income-related* horizontal equity requires that *all income groups* convert the same fraction of their entitlements into actual LTC use.[3]

[2] Consistently, in Section 3 we have defined need-predicted use (i.e. use that we would observe if the influence of non-need factors were neutralized) as: $y_i^N = \frac{\bar{y}}{\bar{x}} x_i$.

[3] In a previous version of our analysis (Tenand et al., 2018), we imposed coefficient β^N to be equal to 1. This meant that we measured horizontal inequity as the deviation from a situation in which not only all individuals with the same entitlements have the same LTC use, but also average use equals average

Assumption (2) reflects the fact that within our study population (only those eligible for LTC in 2012) and the functioning of the Dutch LTC system, LTC use is necessarily zero when entitlements are zero.

Contributions of non-need factors to HI

In the decomposition formula, the contribution of a given factor to inequality is all the larger as its partial correlation with LTC use is high and that is is unequally distributed across the income distribution.

As it derives from a linear regression without ruling out all sources of endogeneity, the contribution of each variable needs not be causal (Fleurbaey and Schokkaert, 2011; van Doorslaer and van Ourti, 2011). Yet such a decomposition can provide some useful insights into the potential sources of inequity and guide further investigation.[4] We thus use the available socio-demographic information as non-need factors to perform the regression underlying the decomposition. By doing so, we do not assume that age and gender, in particular, are not legitimate determinants of LTC use: given that we control for needs in the regression, we instead take that age and gender (and all the other non-need factors) should not weigh in the use of LTC services *above and beyond* the assessment done by CIZ.

In order to interpret the contribution of a variable z_k, one must combine the descriptive statistics to get $\bar{z}_k$, the estimates of the linear regression (Table B.1) to get $\hat{\beta}_k^{NN}$, and the concentration index $CI(z_k)$ of the variable provided in Table B.2.

B.3 Linear estimates

Table B.1 reports the estimates $\hat{\beta}_k^{NN}$. Column (1) reports the estimates obtained on the entire sample, while Columns (2) and (3) report the estimates obtained on the subgroup eligible for home care and institutional care respectively.

For better readability, we report the coefficients associated with the income, wealth and regional dummies separately (Figures B.1 to B.9).

entitlements. If we were to impose a 1-1 relationship between entitlements and care use on average and measure horizontal inequity by the deviation from this situation, as in Tenand et al. (2018), we believe that we would pick up both *horizontal* inequity and *vertical* inequity in LTC use. As a matter of fact, the way that $HI(y)$ was defined in in Tenand et al. (2018) implied that it could be different from zero even in the case all individuals would all consume the exact same proportion of their entitlements.

[4]In particular, one potential concern is that income and wealth could be lowered by a high use of LTC services. We believe there is little scope for a reverse causality bias here: income being mostly made of pensions, it is independent from the disability status of the individuals. As individual co-payments are capped, the medium-run impact of intensive LTC use on wealth is limited. We also exclude individuals with mental health issues and handicaps, which may affect life-time earnings.

Table B.1: Model of LTC use: linear regression results, for the entire sample and by subgroups.

	Dependent variable: value of LTC use		
Eligible for:	Entire sample	Home care	Institutional care
	(1)	(2)	(3)
Need variable			
CIZ-assessed LTC needs	0.739	0.538	0.864
Non-need variables			
Age: 60-69	-3.166***	-1.240***	-3.500***
	(0.078)	(0.070)	(0.128)
Age: 70-79	-1.706***	-0.710***	-1.880***
	(0.057)	(0.052)	(0.082)
Age: 85-89	1.036***	0.503***	0.619***
	(0.057)	(0.056)	(0.070)
Age: 90+	2.763***	1.458***	1.692***
	(0.063)	(0.077)	(0.071)
Gender: woman	0.029	0.559***	-0.722***
	(0.045)	(0.044)	(0.059)
Having a partner in the household	-4.917***	-1.441***	
	(0.058)	(0.059)	
Number of household members	0.785***	-0.009	
	(0.041)	(0.041)	
Married			-1.281***
			(0.065)
Origin: foreign Western country	-0.571***	-0.400***	-0.086
	(0.075)	(0.072)	(0.098)
Origin: Turkey	-6.144***	-2.665***	-3.927***
	(0.237)	(0.173)	(0.704)
Origin: Marocco	-4.978***	-1.344***	-4.634***
	(0.310)	(0.247)	(0.874)
Orign: Suriname	-3.283***	-0.989***	-0.040
	(0.246)	(0.227)	(0.372)
Orign: Dutch Caribbean	-1.634***	0.327	-0.365
	(0.485)	(0.477)	(0.623)
Origin: other non-Western country	-4.915***	-2.459***	-2.125***
	(0.304)	(0.261)	(0.480)
Owner of her house	-1.353***	-0.218**	-1.561***
	(0.073)	(0.073)	(0.089)
Dummies for income deciles	$p < 0.01$	$p < 0.01$	$p < 0.01$
Dummies for wealth deciles	$p < 0.01$	$p < 0.01$	$p < 0.01$
Dummies for LTC contracting regions	$p < 0.01$	$p < 0.01$	$p < 0.01$
Observations	616934	401262	287932

NOTES: Huber-White robust standard errors in parentheses; * $p < 0.10$, ** $p < 0.05$, *** $p < 0.01$. The monetary value of LTC use and CIZ-assessed needs, income and wealth are expressed in thousands euros.

Figure B.1: Estimates of the coefficients of income deciles, entire sample.

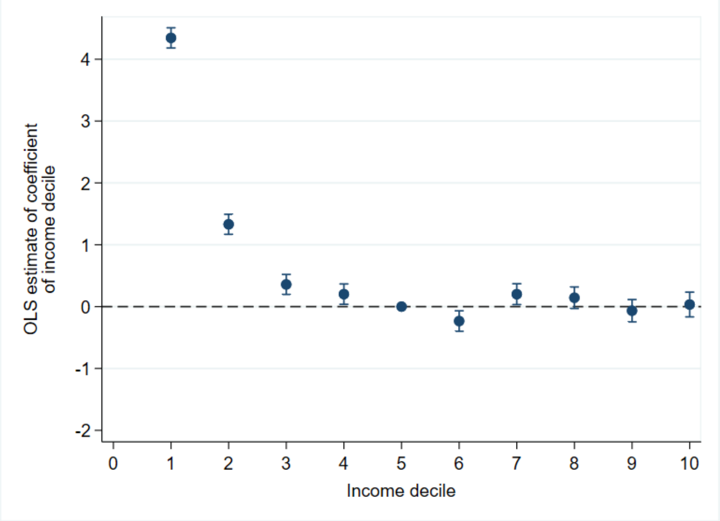

NOTES: For each coefficient, the 5% confidence interval is depicted (computed using the Huber-White robust standard errors). LTC use is expressed in monetary value, in thousands euros per year. Individuals are ranked by their 2011 disposable income. Estimates from the OLS regression (Equation (2) and Column (1) of Table B.1).
READING: Being in the 2nd bottom income decile is associated with a higher use of LTC by €2,500, compared to belonging to the 5th income decile.

Figure B.2: Estimates of the coefficients of wealth deciles, entire sample.

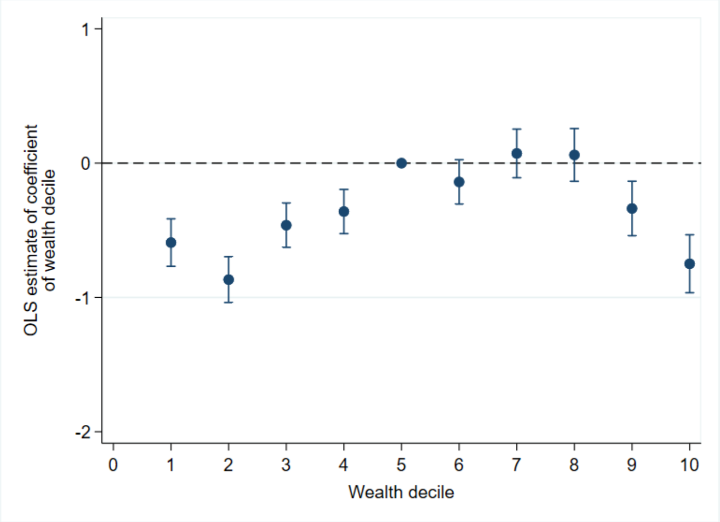

NOTES: For each coefficient, the 5% confidence interval is depicted (computed using the Huber-White robust standard errors). LTC use is expressed in monetary value, in thousands euros per year. Individuals are ranked by their 2011 per capita household wealth. Estimates from the OLS regression (Equation (??) and Column (1) of Table B.1).
READING: Being in the 10th wealth decile is associated with a lower use of LTC by €500, compared to belonging to the 5th wealth decile.

Figure B.3: Estimates of the coefficients of LTC contracting regions, entire sample.

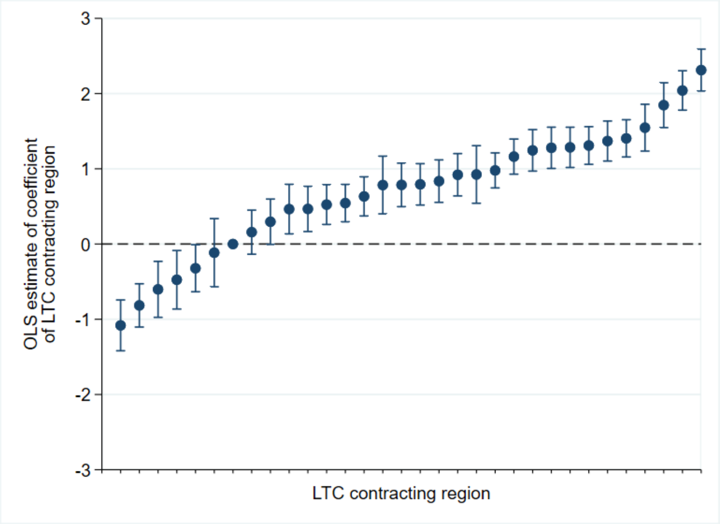

Notes: For each coefficient, the 5% confidence interval is depicted (computed using the Huber-White robust standard errors). LTC use is expressed in monetary value, in thousands euros per year. Ranking of regions from the left to the right is made according to the value of their coefficient. Estimates from the OLS regression (Equation (2) and Column (1) of Table B.1).
Reading: Living in the region ranked eight from the left is associated with a higher LTC use of about €1,500, compared to living in the region ranked first from the left.

Figure B.4: Estimates of the coefficients of income deciles, subgroup eligible for home care.

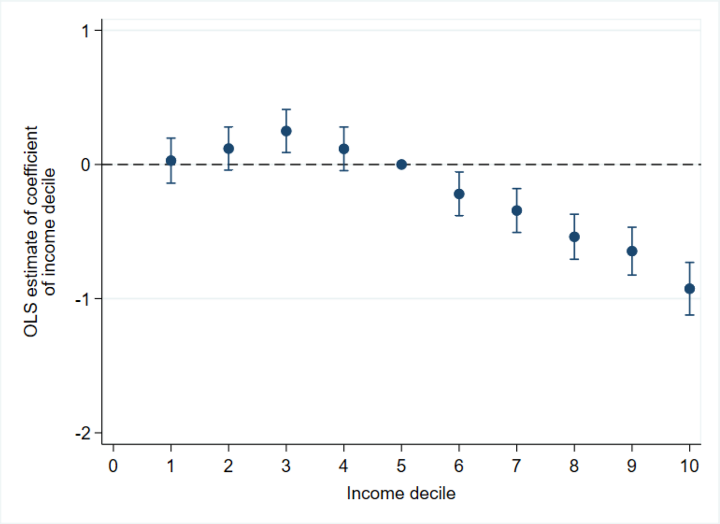

Notes: For each coefficient, the 5% confidence interval is depicted (computed using the Huber-White robust standard errors). LTC use is expressed in monetary value, in thousands euros per year. Individuals are ranked by their 2011 disposable income. Estimates from the OLS regression (Equation (2) and Column (2) of Table B.1).

Figure B.5: Estimates of the coefficients of wealth deciles, subgroup eligible for home care.

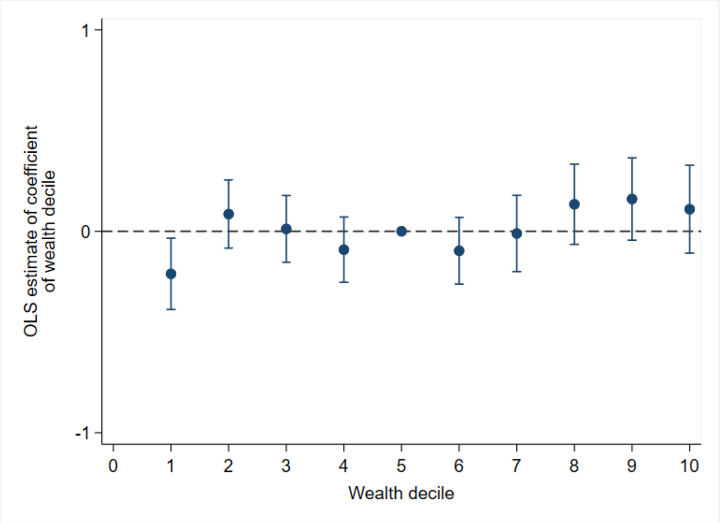

Notes: For each coefficient, the 5% confidence interval is depicted (computed using the Huber-White robust standard errors). LTC use is expressed in monetary value, in thousands euros per year. Individuals are ranked by their 2011 per capita household wealth. Estimates from the OLS regression (Equation (2) and Column (2) of Table B.1).

Figure B.6: Estimates of the coefficients of LTC contracting regions, subgroup eligible for home care.

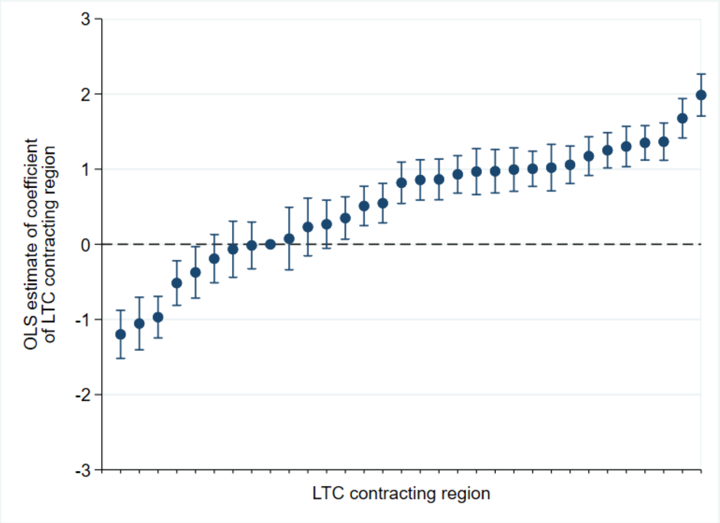

Notes: For each coefficient, the 5% confidence interval is depicted (computed using the Huber-White robust standard errors). LTC use is expressed in monetary value, in thousands euros per year. Ranking of regions from the left to the right is made according to the value of their coefficient. Estimates from the OLS regression (Equation (2) and Column (2) of Table B.1).

Figure B.7: Estimates of the coefficients of income deciles, subgroup eligible for institutional care.

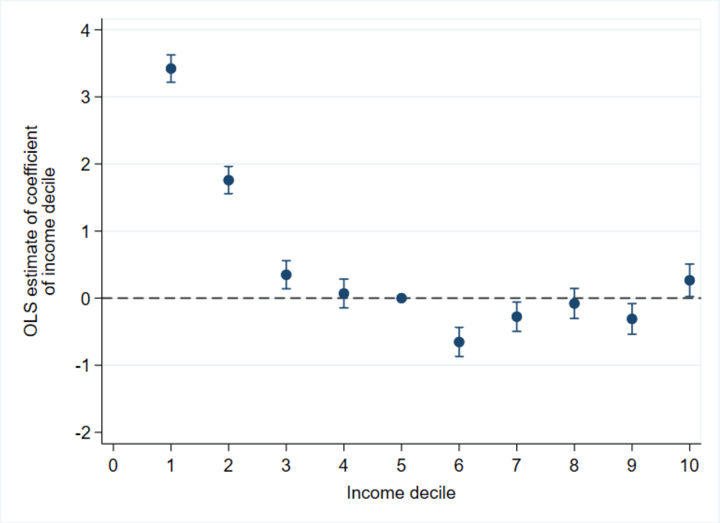

Notes: For each coefficient, the 5% confidence interval is depicted (computed using the Huber-White robust standard errors). LTC use is expressed in monetary value, in thousands euros per year. Individuals are ranked by their 2011 disposable income. Estimates from the OLS regression (Equation (2) and Column (3) of Table B.1).

Figure B.8: Estimates of the coefficients of wealth deciles, subgroup eligible for institutional care.

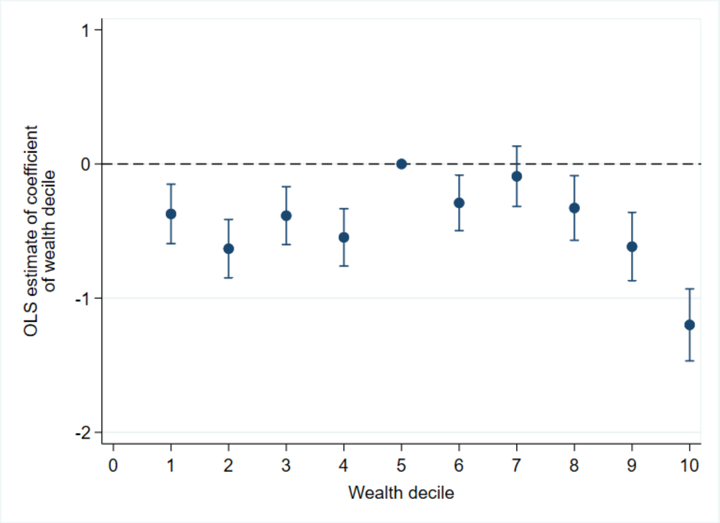

Notes: For each coefficient, the 5% confidence interval is depicted (computed using the Huber-White robust standard errors). LTC use is expressed in monetary value, in thousands euros per year. Individuals are ranked by their 2011 per capita household wealth. Estimates from the OLS regression (Equation (2) and Column (3) of Table B.1).

Figure B.9: Estimates of the coefficients of LTC contracting regions, subgroup eligible for institutional care.

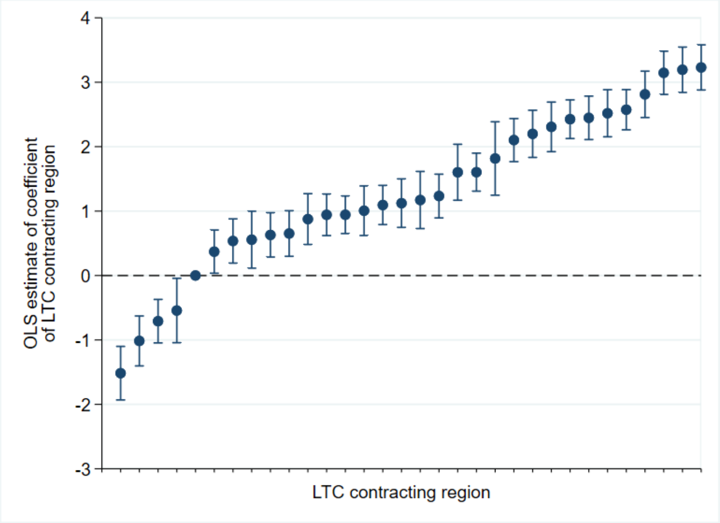

NOTES: For each coefficient, the 5% confidence interval is depicted (computed using the Huber-White robust standard errors). LTC use is expressed in monetary value, in thousands euros per year. Ranking of regions from the left to the right is made according to the value of their coefficient. Estimates from the OLS regression (Equation (2) and Column (3) of Table B.1).

B.4 Concentration indices of CIZ-assessed needs and non-need factors

Table B.2: Concentration indices of CIZ-assessed needs and non-need factors.

	Entire sample	Eligible for:	
		Home care	Institutional care
	(1)	(2)	(3)
CIZ-assessed LTC needs	-0.0358	0.0136	-0.0260
Age	-0.0080	-0.0064	-0.0058
Woman	-0.0460	-0.0434	-0.0435
Having a partner in the household	0.1195	0.0723	–
Number of household members	0.0780	0.0609	–
Married	–	–	0.0716
Origin: foreign Western country	0.0454	0.0422	0.0505
Origin: Turkey	-0.3813	-0.4352	-0.3392
Origin: Morocco	-0.4107	-0.4752	-0.3441
Origin: Surinam	-0.3033	-0.3217	-0.3261
Origin: Dutch Caribbean	-0.3146	-0.3098	-0.3571
Origin: other non–Western country	-0.3731	-0.4242	-0.3226
Disposable income	0.2592	0.2539	0.2601
Per capita household wealth	0.3857	0.3760	0.3966
Home owner	0.2941	0.2498	0.3463

Notes: When estimating the model of LTC use on the subgroup of individuals eligible for institutional care, we do not include the household composition as a control variable. In addition, we replace the dummy "having a partner in the house" by the marital status.

A negative (positive) concentration index indicates that the characteristic is relatively more (less) widespread among the income-poor than among the rich. For example, Table B.2 indicates that women and non-Western migrants tend to be poorer, while home owners and individuals with a partner tend to be richer.

C Robustness

C.1 Excluding individuals who died in 2012

Figure C.1: Probability to have died in 2012, by income decile.

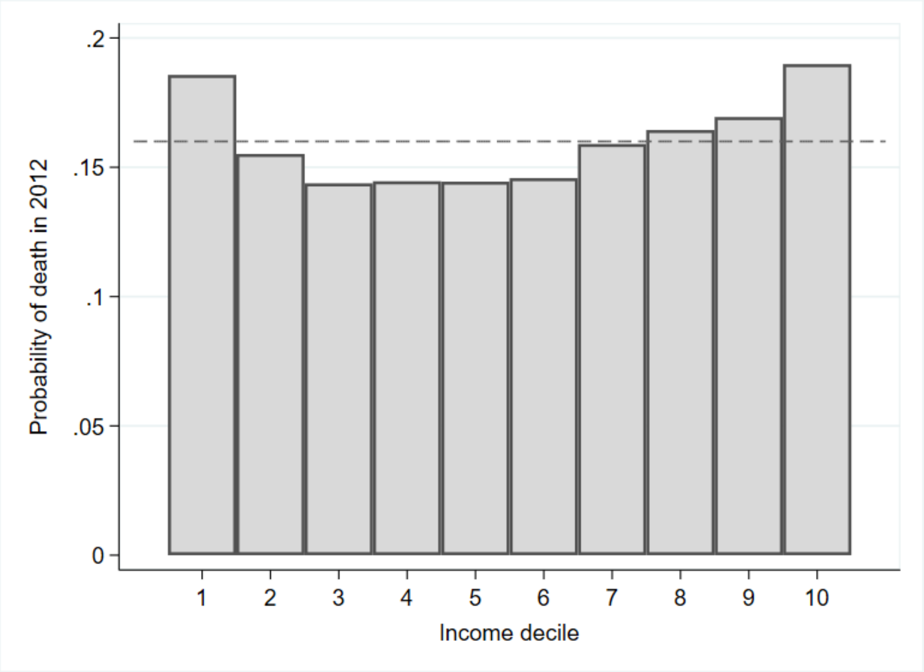

SAMPLE: Individuals 60 and older eligible for public home care in the Netherlands in 2012 due to a somatic or psycho-geriatric condition (N=616,934).
NOTES: The dashed horizontal line indicates the sample one-year mortality rate.

Table C.1: Concentration and horizontal inequity indices: Excluding the deceased (entire sample)

	CI (1)	C^N (2)	*HI* (3)	N
Entire sample				
Baseline	-0.0596***	-0.0254***	-0.0342***	616,934
Excluding the dead	-0.0670***	-0.0320***	-0.0350***	518,097
Eligible for home care				
Baseline	-0.0239***	0.0027***	-0.0267***	401,262
Excluding the dead	-0.0395***	-0.0125***	-0.0270***	348,702
Eligible for institutional care				
Baseline	-0.0392***	-0.0179***	-0.0213***	287,932
Excluding the dead	-0.0432***	-0.0186***	-0.0245***	227,251

C.2 Wealth-related inequalities in LTC use

Figure C.2: Distribution of need-standardized LTC use across wealth deciles: Entire population

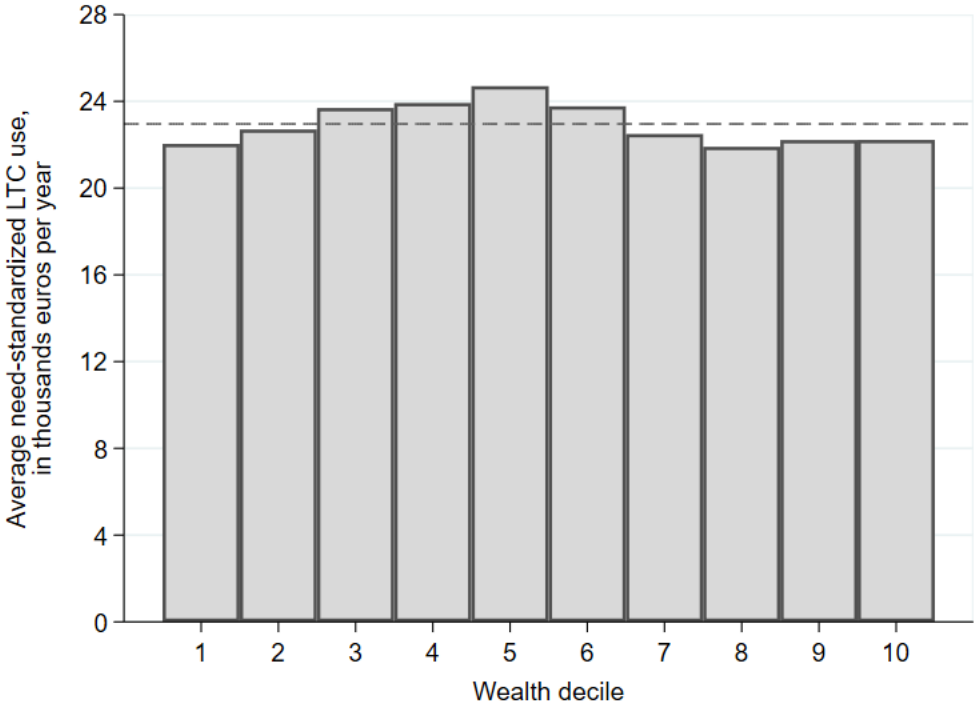

SAMPLE: Individuals 60 and older eligible for public LTC in the Netherlands in 2012 due to a somatic or psycho-geriatric condition (N=616,934).
NOTES: LTC use is expressed in annual monetary value, in thousands euros. The dashed horizontal line represents the average value of LTC use in the sample. Individuals are ranked by their per capita 2011 household wealth.

Figure C.3: Distribution of need-standardized LTC use across wealth deciles, by subgroup.

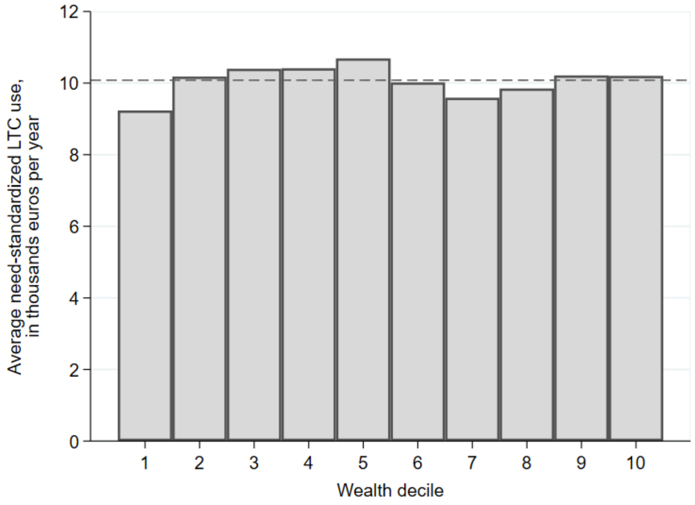

Panel A (top): Individuals eligible for home care.

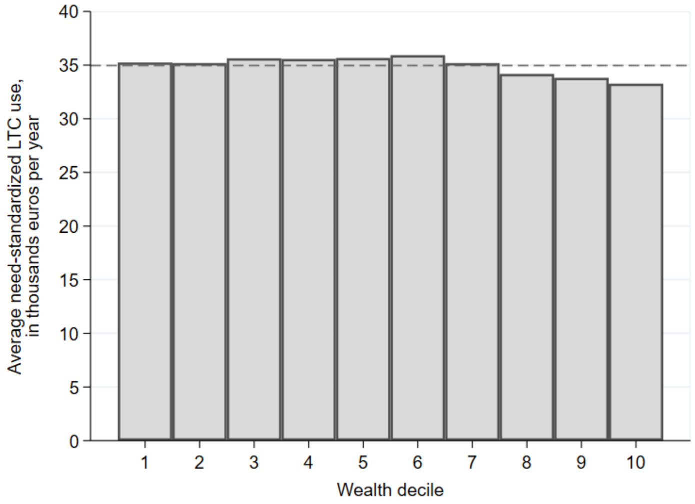

Panel B (bottom): Individuals eligible for institutional care.

Samples: Individuals 60 and older eligible for either public home care (Panel A; N=401,262) or institutional care (Panel B; N=287,932) in the Netherlands in 2012 due to a somatic or psycho-geriatric condition.
Notes: LTC use is expressed in annual monetary value. LTC use is expressed in annual monetary value. In Panel A, it is the sum of the value of home care services used in kind and of the imputed value of LTC vouchers granted while the individual was eligible for home care in 2012. In Panel B, it is the sum of the value of home care services used in kind, of the value of LTC vouchers granted and of elderly institutional care received in 2012. Individuals are ranked by their per capita 2011 household wealth.

D Inference

Standard error of the concentration index of use

In order to derive standard errors on $CI(y)$, we use the convenient regression (O'Donnell et al., 2008). The convenient regression (Kakwani et al., 1997) allows to derive the concentration index directly from the estimation of the regression of a transformation of the LTC use variable on the fractional rank in the income distribution. The convenient regression corresponds to the following specification:

$$2\sigma_r^2(y_i/\mu) = \alpha + \delta r_i^I + \epsilon_i \tag{9}$$

where σ_r^2 is the variance of the fractional rank. The OLS estimate of δ corresponds to the concentration index of y.

However, the standard error associated to δ does not incorporate the sampling variability of the dependent variable in Equation 9 (which contains an estimate of the population mean of LTC use, μ). The solution is to regress the un-transformed outcome, y, on the fractional rank, then transform the coefficient on the fractional rank, and apply a delta method to derive a correct standard error:

$$y_i = \alpha_1 + \delta_1 r_i^I + u_i \tag{10}$$

The estimate of the concentration index CI is then equal to:

$$\hat{\delta} = \Big(\frac{2\sigma_r^2}{\mu}\Big)\hat{\delta}_1$$

This expression can be rewritten as:[5]

$$\hat{\delta} = \Big(\frac{2\sigma_r^2}{\hat{\alpha}_1 + \hat{\delta}_1/2}\Big)\hat{\delta}_1$$

The estimate of the concentration index is now written as a function of the regression coefficients from Equation 10; we can then apply the delta method to derive the standard error of the concentration index.[6]

[5]Using the fact that the sample mean OLS predicted value of the outcome is by construction equal to the mean of the outcome, μ, and that it is also equal to the predicted outcome at the sample mean of the fractional rank. The sample mean of the fractional rank is simply equal to 0.5.

[6]In Stata, this can be done using the command `nlcom` (O'Donnell et al., 2008).

Standard error of the concentration of needs

We have used the convenient regression approach to derive the standard error of $C^N(y)$, by replacing y_i by x_i in Equation (10).

Standard error of the horizontal inequity index

We can derive the horizontal inequity index as the concentration index of the (indirectly) need-standardized LTC use, y_i^{IS} (cf. Section 3 of the paper). The standard error of $HI(y)$ is obtained again by using the convenient regression approach: we replace y_i by $y_i^{IS} = y_i - (\bar{y}/\bar{x})x_i + \bar{x}$ in Equation (10).

E Additional descriptive statistics

E.1 Income, wealth, LTC entitlements and use

Table E.1: Additional descriptive statistics on the distribution of income, wealth, LTC entitlements and use

	Entire study population		Sub-population eligible for home care		Sub-population eligible for institutional care	
	1st ptile	*99th ptile*	*1st ptile*	*99th ptile*	*1st ptile*	*99th ptile*
Equivalized income	9,796	83,171	11,182	84,055	9,459	80,876
Wealth	0	1,028,265	0	1,060,988	0	982,411
LTC entitlements	209	126,309	122	151,180	1,056	76,777
LTC use	0	94,873	0	94,824	0	87,087

NOTES: Ptile stands for percentile. Income is expressed in euros over year 2011. Wealth is expressed in euros and measured on December, 31st 2011. Care use and entitlements are experessed in euros over year 2012.

E.2 Take-up of LTC vouchers by income deciles

Table E.2: Take-up and imputed value of LTC vouchers, by income (entire study population)

	Decile									
Take-up (% with any voucher used)	4.7	5.1	3.4	3.0	2.8	3.2	3.7	4.5	5.7	7.9
Imputed value of vouchers used (% of total care used)	4.4	4.7	3.0	2.7	2.5	2.8	3.2	4.0	5.0	7.0

References

College voor Zorgverzekeringen (2012). *Zelf uw zorg inkopen in 8 stappen 2012.*

Fleurbaey, M. and Schokkaert, E. (2011). Equity in Health and Health Care. In Pauly, M. V., Mcguire, T. G., and Barros, P. P., editors, *Handbook of Health Economics*, volume 2, pages 1003–1092. Elsevier.

Kakwani, N., Wagstaff, A., and van Doorslaer, E. (1997). Socioeconomic inequalities in health: Measurement, computation, and statistical inference. *Journal of Econometrics*, 77(1):87–103.

Nederlandse Zorgautoriteit (2011a). *Prestatiebeschrijvingen en tarieven zorgzwaartepakketten.*

Nederlandse Zorgautoriteit (2011b). *Tarieven en Prestaties - Tariefbeschikking extramurale zorg (2e/3e compartiment) Nummer: 502-12-1.*

O'Donnell, O., van Doorslaer, E., and Wagstaff, A. (2012). Decomposition of Inequalities in Health and Health Care. In *The Elgar Companion to Health Economics, Second Edition*, Economics 2012, pages 179–191. Andrew Jones, Edward Elgar Publishing Limited edition.

O'Donnell, O., van Doorslaer, E., Wagstaff, A., and Lindelow, M. (2008). *Analyzing Health Equity Using Household Survey Data: A Guide to Techniques and their Implementation.* The World Bank.

Tenand, M., Bakx, P., and van Doorslaer, E. (2018). Equal long-term care for equal needs with universal and comprehensive coverage? an assessment using dutch administrative data. Tinbergen Discussion Paper 18-098V, Tinbergen Institute.

van Doorslaer, E. and van Ourti, T. (2011). Measuring inequality and inequity in health and health care. In *The Oxford Handbook of Health Economics.* Sherry Glied and Peter C. Smith, Oxford University Press edition.

Wagstaff, A., van Doorslaer, E., and Watanabe, N. (2003). On decomposing the causes of health sector inequalities with an application to malnutrition inequalities in Vietnam. *Journal of Econometrics*, 112(1):207–223.

The present list mentions only the references quoted in the supplementary materials.
